# Supplementary material for: Rural active aging as a development strategy: Nexus of physical exercise participation and multidimensional poverty in China
Source: PLoS One. 2025 Sep 2;20(9):e0331199. doi: 10.1371/journal.pone.0331199 (PMC12404369; doi:10.1371/journal.pone.0331199)
Supplement: Attachment — Submitted filename: Response letter.pdf [file pone.0331199.s004.pdf]

Rural Active Aging as Development Strategy: Quantifying Exercise's Multidimensional Poverty Reduction Effects

Qi-fei Xia[1], Shu Xuan[2], Guo-you Qin[3*]

1 School of Physical Education, Ankang College, Ankang, China

2 School of Physical Education, Hunan University, Changsha, Hunan, China

3College of Physical Education, Hanjiang Normal University, Shiyan, China

* Correspondence: Prof. Guo-you Qin,

Email: qinguoyou22@163.com

Response Letter to Reviewer's Comments

Comments from the Editor:

Thank you for submitting your manuscript to PLOS ONE. After careful consideration, we feel that it has merit but does not fully meet PLOS ONE's publication criteria as it currently stands. Therefore, we invite you to submit a revised version of the manuscript that addresses the points raised during the review process.

Reponse: We thank all the editors and reviewers for their valuable comments and suggestions.-We have carefully revised the manuscript to enhance its clarity and facilitate the understanding of the readers. Our point-to-point responses are presented in the following We-hope that the revision would satisfactorily address the comments and concerns of the editors and reviewers.

Response Letter

Dear Editor:

Thank you for your letter and comments concerning our manuscript entitled "Rural Active Aging as Development Strategy: Quantifying Exercise's Multidimensional Poverty Reduction Effects" (Manuscript ID: PONE-D-25-1951), Those comments are all valuable and very helpful for revising and improving our paper. According to the Editor/Reviewer's comments. we have made extensive modifications to our manuscript. In this revised version. Our response is given in normal front and changes/additions to the manuscript are given in the red text. the detailed corrections are listed below.

I hope that the revised manuscript for publication in *PLOS One*. Thank you again for your help with the manuscript. I am more than happy to provide any further information you may need.

Responses to Reviewer # 1:

Q1: Abstract

The abstract requires a complete rewrite. The current structure, which follows subheadings like Introduction, Methods, Results, Conclusion, and Recommendations, is not appropriate for this journal. Moreover, Propensity Score Matching (PSM) is mentioned under results but not reflected in the methos. All methods used should be clearly outlined along with their purpose and relevance to the study.

ANSWER: We sincerely appreciate the valuable comments. According to your suggestions, we have rewritten the abstract section with the following main modifications: firstly, we have deleted the subheadings of Introduction, Methods, Results, Conclusions and Recommendations to comply with the journal standards; secondly, we have added the main results of the robustness testing methods in the Methods section, and especially in the Propensity Score Matching method we have produced an ATT effects table, which enables us to have a clearer grasp of the empirical process of our data analyses Thirdly, all of our methods are used to show the relevance of the purpose and the study. We hope our revisions address your concerns. **Specific revisions can be found on page 1 of the manuscript, lines 11-33.**

Q2：Additionally, there are inconsistencies in the figures presented for the indirect effects between the document and the submission system. Most importantly, the results section lacks clarity. Only results directly linked to the study's objectives

should be presented; currently, the inclusion of mediating factors, moderating variables, and heterogeneous effects lacks coherence and alignment. As it stands, the presentation in the abstract is unclear and disjointed without clearly showing the mechanism.

ANSWER: Thanks for the comment. Following your suggestions, we have revised the presentation of the results of the mediating effects, moderating effects and heterogeneity analyses in the Abstract section, and in the Indirect Effects section, we have focused on re-adopting the KHB method to validate the indirect effects and what our purpose was in using this method. In the abstract section, readers can more clearly understand that we are showing the purpose of the study through a more direct and coherent presentation. We hope our revisions address your concerns. **Specific revisions can be found on page 1 of the manuscript, lines 11-33.**

Q3:Introduction

This version of the introduction is more refined than the previous submission. However, there are still significant gaps:

Economic Rationale of Physical Exercise: The paper does not adequately explain how physical exercise contributes to income premiums. There is a need to clarify whether physical exercise incurs financial costs —potentially reducing disposable income for the elderly —or whether it is a free public service that can improve health and reduce medical expenditures. This nuance is essential in understanding the role of exercise in

addressing multidimensional poverty, which extends beyond health to encompass education and overall quality of life. The authors are encouraged to reference relevant literature to build a stronger argument for this connection. Moreover, Are the exercises studied communal or individual in nature?

ANSWER: Thanks for the comment. We are very grateful that you have carefully read our manuscript and made valuable suggestions for our introduction part. In this section, we have made careful modifications, reviewed and added relevant literature: the specific modifications are as follows: Firstly, we have added the impact of physical activity on the healthcare costs of middle-aged and elderly people, specifically, the income premium of physical activity in the case of middle-aged and elderly people in terms of disposable income through the promotion of health management and the saving of healthcare costs. Second, as multidimensional poverty is a composite indicator, we also added a reference to physical activity developing the physical ability to maintain vitality, mastery, pursue social interactions, and live independently, providing a fuller rationale for enhancing the link between physical activity and multidimensional poverty. Thirdly, the reviewer mentioned whether the study's exercise is collective or individual in nature, we explain as follows: this study adopts the Chinese Family Tracking Survey (CFPS) data, which is a micro-survey data focusing more on individual physical activity, and the data variable responses are self-reported by the respondents, if we want to investigate the collective nature of the

survey, the CFPS data should include whether to participate in sports clubs, sports social organisations and other responses, we will pay more attention to physical activity in the subsequent study. In subsequent studies we will pay more attention to the use of multilayered nested models to validate the impact of physical activity on community/collective multidimensional poverty and to supplement relevant research evidence. **See lines 70-86 on page 4 of the manuscript for specific revisions.**

Added references

Liu, T., Yao, Y., Yang, Z., Li, K., Yu, T., & Xia, Y. (2024). The crowding-out effect of physical fitness activities on medical expenditure in the aged group. Frontiers in Public Health, 12, 1425601.

Bergland, A., Fougner, M., Lund, A., & Debesay, J. (2018). Ageing and exercise: building body capital in old age. European Review of Aging and Physical Activity, 15, 1-9.

Q4: Policy Context – National Fitness and Healthy China Strategies: A brief but clear elaboration on the National Fitness and Healthy China strategies would enhance the introduction by grounding the study within the national policy framework. This background is critical for understanding why physical exercise is a focus in the first place and how it ties into broader goals of poverty reduction and well-being.

ANSWER: Thanks for the comment. Following your suggestions, we have added a short description of the national policy in the background section of the manuscript and the gap between rural sport and urban development in this context, to better explain the current importance of fitness for all for

physical activity, and to further explore the relationship between physical activity and multidimensional poverty in rural old age, which can better serve the goal of active ageing. **See lines 71-80 on page 4 of the manuscript for specific revisions.**

Added references

Xinhua News Agency. The central committee of the communist party of china and the state council issued the 'Healthy China 2030' Planning Outline; 2016. Available from: http://www.gov.cn/zhengce/2016-10/25/content_5124174.htm(open in a new window). Accessed June 13, 2025.

Q5：Mediating and Moderating Variables – Contextual Clarity: The roles of social capital (the value of an individual's position within organizational structures) and cultural capital (the precondition for engaging in aesthetic and recreational activities, often tied to educational background) are introduced, but their relevance is not clearly explained. Key questions remain unanswered: What is the mechanism through which these forms of capital mediate the relationship between physical exercise and multidimensional poverty? Similarly, the moderating roles of internet access and public service provision must be grounded in a contextual framework. It is not enough to say these factors were previously unconsidered; authors must justify their inclusion based on theoretical or empirical reasoning, showing how these variables interact with the core relationship under study. Context and conceptual connection

are essential for establishing academic and practical relevance. In short why are they important in this study? What is the background?

ANSWER: Thanks for the comment. Thank you very much for your valuable suggestions. In the background section, we have rewritten it with the following main changes: in terms of mediating mechanisms. We have made theoretical additions and empirical reasoning evidence, based on Bourdieu's theoretical framework, analysed the relationship between rural social capital and multidimensional poverty, and revealed the generative principle of the social capital construct in sport; cultural capital is based on Bourdieu's cultural capital, and we have collected a large amount of empirical evidence to explain the correlation between physical activity → cultural capital → multidimensional poverty. In terms of the moderating mechanism, we have supplemented the evidence from relevant studies and found out how the use of network services and the level of public services as a transforming factor are associated with physical activity and multidimensional poverty based on the viability theory, so as to better reveal the intrinsic moderating relationship. We emphasised the combination of questions and empirical evidence during the revision process to build a rich theoretical-empirical reference for the background and better reveal the influential role between the mechanisms. We hope that our revisions address the reviewers' concerns. Specific revisions can be found on page 4 and lines 87-124 of page 5 of the manuscript.

Added references

1. Zhao, J., Liu, X., Gao, Y., Li, D., Liu, F., Zhou, J., ... & Wang, Y. (2024). Estimates of multidimensional poverty and its determinants among older people in rural China: evidence from a multicenter cross-sectional survey. BMC geriatrics, 24(1), 835.
2. Liang, H., Yue, Z., Liu, E., & **ang, N. (2020). How does social capital affect individual health among the elderly in rural China? — Mediating effect analysis of physical exercise and positive attitude. PloS one, 15(7), e0231318.
3. Xu, H., Zhang, C., & Huang, Y. (2023). Social trust, social capital, and subjective well-being of rural residents: Micro-empirical evidence based on the Chinese General Social Survey (CGSS). Humanities and Social Sciences Communications, 10(1), 1-13.
4. Zhang, W. (2022). Social capital, income and subjective well-being: evidence in rural China. Heliyon, 8(1).
5. Magro - Montañés, B., Pabón - Carrasco, M., Romero - Castillo, R., Ponce - Blandón, J. A., & Jiménez - Picón, N. (2024). The relationship between neighborhood social capital and health from a biopsychosocial perspective: A systematic review. Public Health Nursing, 41(4), 845-861.
6. Oh, E. A., & Bae, S. M. (2023). The relationship between personality, social capital, and life satisfaction of elderly Koreans. Psychogeriatrics, 23(3), 494-502.
7. Zhang, Y., Fang, S., & Huang, J. (2024). Financial inclusion and poverty alleviation: Rethinking theory and intervention strategies based on rural practice in China. Socium, 1, 5-23.
8. Dergance, J. M., Calmbach, W. L., Dhanda, R., Miles, T. P., Hazuda, H. P., & Mouton, C. P. (2003). Barriers to and benefits of leisure time physical activity in the elderly: differences across cultures. Journal of the American Geriatrics Society, 51(6), 863-868.
9. Wang, W., Urakawa, K., & Anegawa, K. (2022). Effects of university graduation on multidimensional poverty risks in Japan. International Journal of Educational Research, 113, 101973.
10. Liang, X., Xiao, H., Hou, F., Guo, X., Li, L., & Huang, L. (2024). Breaking the chains of poverty: Examining the influence of smartphone usage on multidimensional poverty in rural settings. Humanities and Social Sciences Communications, 11(1), 1-17.
11. da Silva, L. H., de Campos, L. W., Chicon, P. M. M., Garces, S. B. B., de Rosso Krug, M., Xavier, A. J., & de Rosso Krug, R. (2022). Effects of a group using digital technologies and physical activity on cognition and health of the elderly. PAJAR-Pan-American Journal of Aging Research, 10(1), e43154-e43154.

12. Laverty, A. A., Webb, E., Vamos, E. P., & Millett, C. (2018). Associations of increases in public transport use with physical activity and adiposity in older adults. International Journal of Behavioral Nutrition and Physical Activity, 15, 1-10.
13. Yue, S. U. N., Tao, H. U. A. N. G., Yanhui, W. A. N. G., & Longfeixue, Y. U. (2022). Multidimensional Poverty-reduction Effect of Rural Basic Public Services in Key Counties for Rural Revitalization. Economic geography, 42(6), 144-155.

Q6:Literature Review

The focus on multidimensional poverty is appreciated, but the literature review leans heavily toward public physical exercise, a focus not clearly emphasized earlier in the manuscript. Are the authors advocating for public/community physical exercise, or is individual exercise also of interest? For instance, how does individual exercise promote social capital in the Chinese rural context?

ANSWER: Thanks for the comment. We are very grateful for your suggestion. In the literature review section, we emphasize the importance of physical activity, but in the first half of the section, we do not distinguish between public and individual physical activity, which we explain as follows: according to the division of physical activity organisation by the Chinese scholar Lu Yuanzhen, there are various forms of physical activity, and according to the organisation of physical activity, it can be classified into individual exercise and group exercise, which we added in the front of the manuscript. In this study, we use microdata and advocate the poverty

reduction path of ‘individual first, public complementary’. In part 1.2 of the manuscript, we focus on the multidimensional indicators of the effects of physical activity on the health, income, life satisfaction and subjective quality of the middle-aged and the elderly, and find that we focus more on the discussion of the individual's behaviour. In rural China, individual exercise has a significant contribution to social capital. For example, when elderly morning exercisers are regularly present in public spaces such as village entrances/ancestral halls (e.g., playing Tai Chi), they become village health landmarks and build weak relational ties. In addition, rural areas in China have significant blood and geographic ties, and physical exercise can establish good trust bond relationships and gain more emotional and informational exchanges. This has been found with sufficient evidence in Liang's (2020) study. See page 9, line 197 of the manuscript for specific changes.

Added references： Liang, H., Yue, Z., Liu, E., & **ang, N. (2020). How does social capital affect individual health among the elderly in rural China?—Mediating effect analysis of physical exercise and positive attitude. PloS one, 15(7), e0231318.

Q7： Additionally, the use of internet services is vague. In Hypothesis H3a, it is unclear whether the internet is used for accessing exercise programs, purchasing

equipment, or social networking. This lack of clarity weakens the theoretical foundation.

ANSWER: Thanks for the comment. According to your comments, we have reviewed a large number of empirical studies. With the continuous advancement of Internet technology, middle-aged and elderly people have more diversified channels to obtain health information, and the Internet can provide middle-aged and elderly people with scientific guidance on exercise and understand the importance of ‘moderate exercise’. In addition, online communities and offline activity organisations can stimulate the cohort effect, which can also enhance the motivation of middle-aged and elderly people to exercise. Currently, there is a large amount of evidence that the Internet is an information tool that allows older adults to choose exercise programmes, purchase equipment or social networks according to their own needs, as shown in the following literature:

1. Guo (2022) used CHARLS data to validate the relationship between Internet use and physical activity among middle-aged and older adults, and emphasised that Internet use is a major source of health information, and that chatting, watching news or videos over the Internet can significantly increase the likelihood of physical activity.

2. Another scholar, Guo (2022), used CFPS data to find that the Internet can promote physical health by increasing the frequency of physical activity

among middle-aged and older adults, a conclusion based on the theory of network gain effect. That is, older people ton access disease prevention and health care knowledge through channels such as WeChat and online publications can increase the frequency of physical activity participation.

All these evidences show the influence of the Internet on physical activity of middle-aged and elderly people, i.e., the Internet as a media tool can enhance the probability of physical activity participation of middle-aged and elderly people.

Added references： 1.Guo, B., Zhang, X., Zhang, R., & Chen, G. (2022). The Association between Internet Use and Physical Exercise among Middle-Aged and Older Adults — Evidence from China. International Journal of Environmental Research and Public Health, 19(24), 16401.

2.Guo, E., Li, J., Luo, L., Gao, Y., & Wang, Z. (2022). The effect and mechanism of Internet use on the physical health of the older people—Empirical analysis based on CFPS. Frontiers in Public Health, 10, 952858.

Q8: Data and Sampling

The decision to define elderly rural residents as individuals aged 45 and above needs stronger justification. Why not 50 or 55? Those aged 45 may have very different physical capacities and socioeconomic contexts compared to those 70 and above. The

empirical strategy must consider these age-related differences, especially given that mediating and moderating effects may differ substantially by age cohort.

ANSWER: Thanks for the comment. Based on the reviewers' suggestions, we explain this as follows: Firstly, we have made reference to the World Health Organisation's classification of different age groups in 2023, in which 45-59 years of age is considered as middle-aged, 60-74 years of age is considered as old-aged, and 75 years of age and above is considered as old-aged with a high life expectancy. This is one of the reference bases for our delineation. In addition, if only the sample of elderly aged 60 and above was selected it would reflect a small sample size and would not be representative. Secondly, in order to address the reviewers' concerns, we supplemented our analyses at the heterogeneity by conducting group regression on different age groups, and found that physical activity had a greater impact on multidimensional poverty among the high-living elderly through the comparison of the differences between the groups, and added accordingly in the Discussion section. I hope this addresses the reviewers' concerns. Specific revisions can be found on page 34, lines 632-640, and lines 830-842 of the manuscript.

Added references：

Li, S., Ye, H., Chen, A., Lan, L., Yang, S., & Ji, F. (2021). Characteristics of hearing loss in elderly outpatients over 60 years of age: an annual cross-sectional study. Acta Oto-Laryngologica, 141(8), 762-767.

Q9: Variables and Methodology

• *Dependent Variable: There is inconsistency in the stated weights applied across the four dimensions of multidimensional poverty. The text claims equal weights, but Table 1 presents unequal weights (1/16 and 1/8). This discrepancy must be resolved.*

ANSWER: Thanks for the comment. According to the reviewer's comments, we make the following explanation: we mainly refer to Song's (2023) way of dividing the four dimensions of multidimensional poverty, which is basically consistent with the existing literature setting. The scholar processed the four dimensions with equal weights, and then the indicators within each dimension were then weighted equally. Table 1 The core principles of indicator weight design vary, the indicator weight formula: single indicator weight = the weight of the dimension to which it belongs / the number of indicators in that dimension, such as the global MPI (Multidimensional Poverty Index) is divided into three major dimensions of health, education, and standard of living, and each of these dimensions has a weight of 1/3, and the number of indicators to set the weight may need to be adjusted:

Dimension	Number of indicators	Individual indicator weights	calculation logic
Healthy	2	1/6	（1/3）/2=0.166
Education	2	1/6	（1/3）/2=0.166

living standards	8	1/24	（1/3）/8=0.0416

National Bureau of Statistics of China (MPI)

Dimension	Number of indicators	Weight	Design logic
Healthy	Medical insurance	1/12	Total dimension weight 1/4
	major illness	1/12	
Education	educational attainment	1/8	Total dimension weight 1/4
	School-age children out of school	1/8	

living standards	Drinking water, sanitation	1/24 each	Total dimension weights 1/2 (6 indicators)
	Housing, fuel	1/24 each	

Therefore, the core of understanding weighting is to grasp the two levers of ‘dimension weighting’ and ‘number of indicators’, which together determine the difference in the final value. I hope this will address the concerns of the reviewers.

Added references：1.Song Y, Cheng Z, Zhou G, et al. Can the rural low income insurance system alleviate multidimensional poverty among the elderly--an empirical analysis based on data from the China Household Tracking Survey. Social Security Research. 2023; (01): 25-40. https://kns.cnki.net/kcms2/article/abstract?v=1i9K2Vab0pOlGC4q5--6iINOm4eETlcLw0PyK-vxnP0PUsf8LdjDTBz9uAbzOjPlz6h0VNRjPmrUACQopKaMktcHBXnPa48-VhJ20k02lqdlskpFfbhKtaKGk6NyADPySzNZuq40078aOG85kZekOudQ4Ui9uirPq8ofRKNEdyxN_mV2uBB7HD11_AqlB3MA&uniplatform=NZKPT

• *Community Social Capital: Authors should clarify how the composite indicator was transformed into a binary variable and why both individual and community levels of social capital are used.*

ANSWER: Thanks for the comment. Thank you very much for your valuable suggestions. We have supplemented the social capital transformation dichotomous variables according to your suggestions, we have explained the answer options for the three questions of collective social capital reciprocity, trust and organisational participation, as well as how to assign them as dichotomous variables, and standardised the individual social capital and collective social capital to get the final assigned variables. In the selection of social capital variables, we reviewed relevant literature and empirical research, the theoretical level, social capital is divided into micro, meso and macro levels: micro-level social capital research representative of the scholar Bourdieu, social capital is defined as ‘the sum of actual and potential resources possessed by members of a social network or group’. Coleman, a representative of the study of social capital at the meso level, believes that social capital is a substance that ‘consists of aspects of the social structure that facilitate specific behaviours of actors’. Putnam, who represents the study of social capital at the macro level, defines social capital as ‘certain features of social organisation, such as trust, norms and networks, that enhance social efficiency by facilitating cooperative action’. On the empirical

level, we refer to the variable construction ideas of Huang (2024), who provides a good paradigm of ideas by transforming the micro level as individual social capital, and the meso and macro levels as collective social capital, together into social capital variables. See lines 399-426 on page 21 of the manuscript for specific revisions.

Added references: HUANG Qian,WANG Qilong,FENG Shunan,et al. Sports Participation, Social Capital and Individual Welfare Enhancement-A Study Based on Welfare Economics Theory and CFPS Survey Data[J]. Research on Physical Education,2024,38(03):1-12.DOI:10.15877/j.cnki.nsic.20240613.001.

• *Equation 12: The interaction term is discussed in the text but not shown in the actual equation. Furthermore, the manuscript lacks clear specification of the models used to estimate the relationships. A dedicated section explaining model choice and their suitability is necessary.*

ANSWER: Thanks for the comment. According to the reviewer's suggestion, we explained the way of constructing the interaction term as well as the purpose in the moderating effect model setting section and under what significant condition the model is statistically significant, in the empirical equation, we refer to Σ α i MSi Σ α j Xj as the interaction term of the independent variable and moderating variable, if the interaction term is significant, there is a moderating effect, and the modelling approach is

mainly . referred to the study conducted by Hayes (2017) research approach. See page 26 of the manuscript, lines 495-504, for details of the modification

Added references： Hayes, A. F. (2017). Introduction to mediation, moderation, and conditional process analysis: A regression-based approach. Guilford publications.

• *Model Specification: The manuscript lacks a clear presentation of the models used to estimate the relationships. This is a major omission. Authors must dedicate a section to describe the econometric models applied and explain why they are appropriate. Robustness checks and treatment of endogeneity should also be thoroughly discussed.*

ANSWER: Thanks for the comment. According to the reviewer's suggestion, we have added the corresponding introduction at the estimation model setting days, which is modified as follows: ordinary least squares (OLS) is a linear least squares method for estimating unknown parameters in a linear regression model. OLS selects a set of parameters for a linear function of the explanatory variables by the principle of least squares: minimising the sum of the squares of the residuals between the dependent variable (the value of the predicted variable) and the predictor variable observed in a given data set. The results estimated by the OLS regression model in the case of large samples are more intuitive, economically meaningful, and convenient for presenting the marginal effects of the explanatory variables on the explained

variables, and OLS regression is more likely to deal with endogeneity. Since the dependent variable multidimensional poverty is the dependent variable and the explanatory variable is the categorical variable in this study, the following multiple linear regression model was set up in order to validate the research hypotheses presented in the previous section and to accurately identify the effect of physical activity participation on multidimensional poverty in old age among family farmers:

$$MPI_i = \alpha_0 + \alpha_1 PA_i + \alpha_2 X_i + \delta_i \quad (1)$$

In Eq. (1), MPIi is the dependent variable family farmers' multidimensional poverty status in old age, PAi is the independent variable physical activity participation, Xi is a series of control variables such as gender, age, etc., and δ i is a random perturbation term. We hope our revisions address the reviewers' concerns. See lines 461-472 on page 24 of the manuscript for details of the revisions.

Q10:Results

This section has major conceptual and methodological weaknesses that compromise the credibility of the findings.

Lack of Model Specification: The authors begin the results section without stating what model(s) were used for the initial estimation. It is unclear whether an OLS, probit, logit, or another specification was applied in the first stage of the analysis. The

term "baseline model" is mentioned but not adequately defined, nor is its function in the analysis (i.e., whether it serves as the main specification or as a robustness check). This must be corrected. Without clearly presenting the baseline econometric model, its control variables, and justification, the rest of the analysis becomes difficult to interpret or trust.

ANSWER: Thanks for the comment. As suggested by the reviewers, we have added the initial estimation model formulation at the beginning of the results section and replaced the title of 3.1 with the OLS model regression results to make it clearer which model was used. It should be explained that in statistical formulation, baseline model refers to the underlying reference model used to assess the performance of the target model, which is usually the result of a model that determines the direct effect of X on Y. It is used as a baseline to measure the performance improvement of more complex models. With the modification, readers will be able to better understand the process of adopting the baseline econometric model and better reveal the statistical significance of the remaining variable components. We hope that our revisions address the reviewers' concerns. **See lines 520-521 on page 27 of the manuscript for details of the revisions.**

Q11：Endogeneity and Self-selection Bias Not Addressed: Participation in physical exercise is likely endogenous, as individuals who choose to participate may differ systematically from non-participants in both observable and unobservable characteristics . The paper does not explain how it addresses self-selection bias. While Propensity Score Matching (PSM) is briefly mentioned elsewhere, it is not integrated coherently into the results narrative. If PSM or any other method (e.g., Heckman selection model, IV approach) was used, it must be formally introduced, justified, and connected to the tables showing the main results. Otherwise, the estimates are at high risk of bias.

ANSWER: Thanks for the comment. Based on the reviewers' suggestions, we have modified this section as follows: whether multidimensional poverty among rural older adults is affected by variables such as physical activity participation, and thus is not simply the result of random assignment. In order to effectively reduce the impact of errors due to selection errors, propensity value matching (PSM) was used. Being able to obtain the propensity scores of the control and the new treatment groups by matching propensity values and further implementing comparative analyses, the net effect of the impact after eliminating the selectivity bias (ATT) can be obtained. In addition, we add the calculations of nearest-neighbour matching, kernel matching, and radius matching, linked to the main display of results in Table 10, demonstrating the usefulness of using propensity score matching as a robustness test. Finally, we add reasons for the selection of the instrumental

variables approach, such as the possibility of endogeneity problems between the independent and dependent variables, i.e., correlation of the independent variables with the error terms of the model. This endogeneity problem usually leads to bias and inconsistency in the OLS model estimates, affecting the reliability of the conclusions. Our revisions better present the findings and make them more coherent and logical. **Details of the modifications are given in lines 658-677 on page 36 of the manuscript and in Table 10.**

Q12：Inappropriate and Incomplete Mediation Analysis (Sobel Test): The use of the Sobel test is methodologically flawed: First, the Sobel test assumes a single mediator in the causal pathway. The authors are using two mediators—social capital and cultural capital—which violates this assumption. The Sobel test does not extend to parallel or sequential multiple mediation models, and using it in this context is statistically inappropriate. Therefore, the reported mediation results are highly questionable. Second, even within the (misapplied) Sobel framework, the authors fail to provide the total, direct, and indirect effects for each mediator. These values are critical for interpreting the mediation mechanism. It is unclear how much of the effect of physical exercise on multidimensional poverty is transmitted via each mediator, nor whether the indirect effects are statistically significant.There is also no discussion of the assumptions underlying this test or any robustness checks for the mediation pathways.

ANSWER: Thanks for the comment. We find your suggestion very informative. In order to address your concerns, we have reviewed a large amount of literature on the analysis of mediation effects, there are stepwise regression, Sobel test, Bootstrap test and KHB decomposition method, in our study we used Sobel test for robustness test, although the results obtained are robust, the specific data results are as follows:

<table>
<tr>
<td>Sobel-Goodman Mediation Tests

Coef Std Err Z P>|Z|
Sobel -.00165216 .00035221 -4.691 2.720e-06
Goodman-1 (Aroian) -.00165216 .00035389 -4.669 3.032e-06
Goodman-2 -.00165216 .00035052 -4.713 2.436e-06

Coef Std Err Z P>|Z|
a coefficient = .019978 .003561 5.61016 2.0e-08
b coefficient = -.082699 .00967 -8.55188 0
Indirect effect = -.001652 .000352 -4.69087 2.7e-06
Direct effect = -.013203 .002722 -4.85016 1.2e-06
Total effect = -.014855 .002731 -5.43944 5.3e-08

Proportion of total effect that is mediated: .11122114
Ratio of indirect to direct effect: .12513927
Ratio of total to direct effect: 1.1251393</td>
<td>Sobel-Goodman Mediation Tests

Coef Std Err Z P>|Z|
Sobel -.00497679 .00058541 -8.501 0
Goodman-1 (Aroian) -.00497679 .00058641 -8.487 0
Goodman-2 -.00497679 .00058441 -8.516 0

Coef Std Err Z P>|Z|
a coefficient = .290215 .025522 11.3713 0
b coefficient = -.017149 .00134 -12.8007 0
Indirect effect = -.004977 .000585 -8.50136 0
Direct effect = -.009878 .002724 -3.62661 .000287
Total effect = -.014855 .002731 -5.43944 5.3e-08

Proportion of total effect that is mediated: .33503048
Ratio of indirect to direct effect: .50382832
Ratio of total to direct effect: 1.5038283</td>
</tr>
<tr>
<td>Social capital intermediation effects</td>
<td>Intermediation effects of cultural capital</td>
</tr>
</table>

However, we have not yet taken into account the impact of multiple mediation effects because the Sobel test can only be used for a single mediator variable test, which, as far as the reviewer is concerned, is inappropriate for robustness testing, and a review of the literature literature reveals the following shortcomings of the Sobel test: firstly, low statistical validity; secondly, bias in the estimation of the standard errors; and thirdly, the inability to handle complex mediators, and its inapplicability to the chained multiple mediation models. For this reason, we deleted the Sobel test and mainly used the KHB decomposition method to verify the mediation effect.

Q13：4. Ambiguity Around KHB Decomposition: The authors mention using KHB decomposition, but the presentation lacks transparency and technical rigor: It is unclear how the estimates were derived, what specific models were decomposed, and whether the KHB method was implemented properly given the type of outcome variable used. There is no citation or methodological explanation of the KHB procedure. The authors must specify the model used for KHB decomposition, the variables included, and the interpretation of the decomposed components (confounding, mediation, etc.). Most critically, there is no clear distinction between what the Sobel test results contribute and what KHB decomposition adds. If both methods were used, the rationale for this dual approach must be stated and justified. If KHB replaced the Sobel test, the latter should not be reported at all. The authors are strongly encouraged to revise this section in light of the methodological shortcomings. For proper guidance, they should consult the following methodological literature on mediation analysis and KHB decomposition:

ANSWER: Thanks for the comment. According to your review suggestions, we have reviewed a large amount of relevant literature on mediating effects and KHB decomposition, and we have made the following modifications: firstly, we have added the citation of the KHB decomposition method and the explanation of the method.The KHB method proposed by Karlson (2011), through the decomposition of effects and statistical tests, can decompose both continuous and dispersed variables, and at the same time, it can be used for the comparison of the mediating effects and contribution rates of multiple

mediating variables. The KHB method can compare the contribution rates of multiple mediating variables, and it is applicable to a variety of models such as reg, logit, ologit, probit, etc., which facilitates the testing of the mediating effects of multiple variables and the comparison of their contribution rates. Second, explain the KHB decomposition model. We reviewed the literature and found that the KHB consists of a simplified model: the total effect of physical activity on multidimensional poverty after the inclusion of control variables; a full model: the direct effect of physical activity on multidimensional poverty after the inclusion of mediating variables; an estimation model: the difference between the total effect and the direct effect, i.e., the mediating effect; and the confounding ratio, which is the ratio between the total effect and the direct effect. In the manuscript we have re-entered the data results for each of the KHB model decompositions as well as the confounding ratios, and the shares of single and multiple mediating variables, to more clearly show the results of the study tests. Details of the changes can be found on page 30, lines 570-587 of the manuscript and in Table 6.

Added references： Kohler, U. , Karlson, K. B. , & Holm, A. . (2011). Comparing coefficients of nested nonlinear probability models. Stata Journal, 11(3), 420-438.

We hope that the revisions and improvements can satisfactorily address the concerns of the reviewers. We appreciate for Editor warm work earnestly, and hope that the correction will meet with approval. Once again, thank you very much for your comments and suggestions.
