# Supplementary material for: Rural active aging as a development strategy: Nexus of physical exercise participation and multidimensional poverty in China
Source: PLoS One. 2025 Sep 2;20(9):e0331199. doi: 10.1371/journal.pone.0331199 (PMC12404369; doi:10.1371/journal.pone.0331199)
Supplement: Attachment — Submitted filename: Comment for Authors.pdf [file pone.0331199.s003.pdf]

Comment for Authors - Rural Active Aging as Development Strategy: Quantifying Exercise's Multidimensional Poverty Reduction Effects

First and foremost, I would like to reiterate that the topic is highly relevant; however, the content lacks focus, resulting in an unclear mechanism. The current version of the manuscript raises more questions than it answers. The sections are not coherent and lack intelligent transitions and consistency. Moreover, in this latest version, the authors have not seriously considered my earlier comments, resulting in a submission that is more or less the same as before. It appears that the authors did not fully understand my previous feedback. I now hope that what I unpack here effectively communicates the ideal approach to presentation and structure.

The title, *"Quantifying Exercise's Multidimensional Poverty Reduction Effects,"* is misleading, as the study does not make any substantial effort to quantify such effects.

Regarding the abstract, the authors should avoid citing results in that manner. Please familiarize yourselves with published articles in this journal. Once again, my earlier comments on structure remain unaddressed. What methods were used, and for what specific objectives?

In the introduction, you need to connect physical exercise, social capital, cultural capital, social services, internet services, and multidimensional poverty in an intelligent and logical manner so readers can understand why these elements matter and what the study aims to achieve. Isn't this too much for one paper? These concepts cannot all be captured as simple dummies. This might explain why the paper lacks focus. The authors must keep in mind that the objective—based on the title—is to clearly examine the impact of physical exercise on MPI and its mechanism.

In the literature review, the emphasis on public physical exercise must be removed. Instead, focus only on literature relating to **individual** physical exercise, and connect it to the variables of interest.

Furthermore, I still maintain that the use of "internet services" is vague. What is it used for? The introduction (line 121) states that digital tools—through real-time health monitoring, text-to-speech systems, and smart fitness-sharing devices—create new opportunities for exercise participation, thereby offering novel ways to combat relative poverty. However, it is unclear how this is captured in the methods section, which simply asks, *"Do you use mobile internet?"* and *"Do you use a computer for internet access?"* This is a very broad dummy variable and does not indicate whether older adults are using mobile or computer internet for the purposes outlined in the introduction. There is a need for more robust measurement before referring to these as moderating variables. Given the level of detail, I believe both variables are more suitable for use in heterogeneous analysis, with a recommendation for future studies to build on them.

Regarding the categorization of the aging population, I still insist that the analysis must reflect these differences throughout. Do the mediating variables have consistent mediating effects across all the different age groups?

I am dissatisfied with the authors' response to my comment on Equation 12. It shows a lack of attention to detail and carelessness. Refer to line 535—the interaction term captured there is not included in Equation 12. I was suggesting that the authors include this term. Can the authors explain in which part of Equation 12 they have included $\sum \alpha_i MS_i \sum \alpha_j X$?

Crucially, every article in this journal includes a dedicated section—usually under "Methodology" or "Materials and Methods"—where the authors explain what methods were used and why. For example:

- **2.2 Analytical or Empirical Strategy**

- **2.2.1 OLS**: Explain how this model is estimated and why it was used. Which study objective does it address?
- **2.2.2 PSM**: Why was PSM selected? What are its advantages, and which objective does it address?
- **2.2.3 Mediation Analysis**: Explain why this method was used and its relevance. A graphical depiction (e.g., Fig 1a or 1b) showing the mediation framework would be helpful. If Fig 1a is used, justify the absence of a connection between the mediators and show how this is modeled. If Fig 1b is chosen, clearly model the scenario with one mediator and also the joint mediation of two variables, as done by Zhang, Mishra, Zhu, and Li (2020).

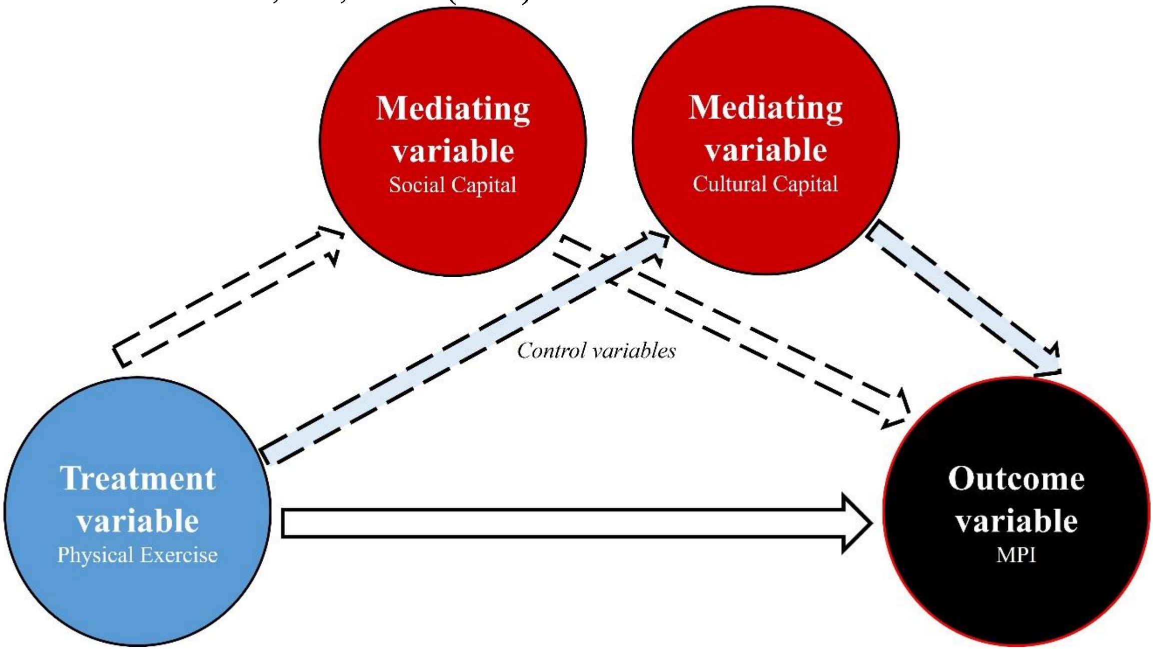

Fig 1a. Without mediating variables connecting

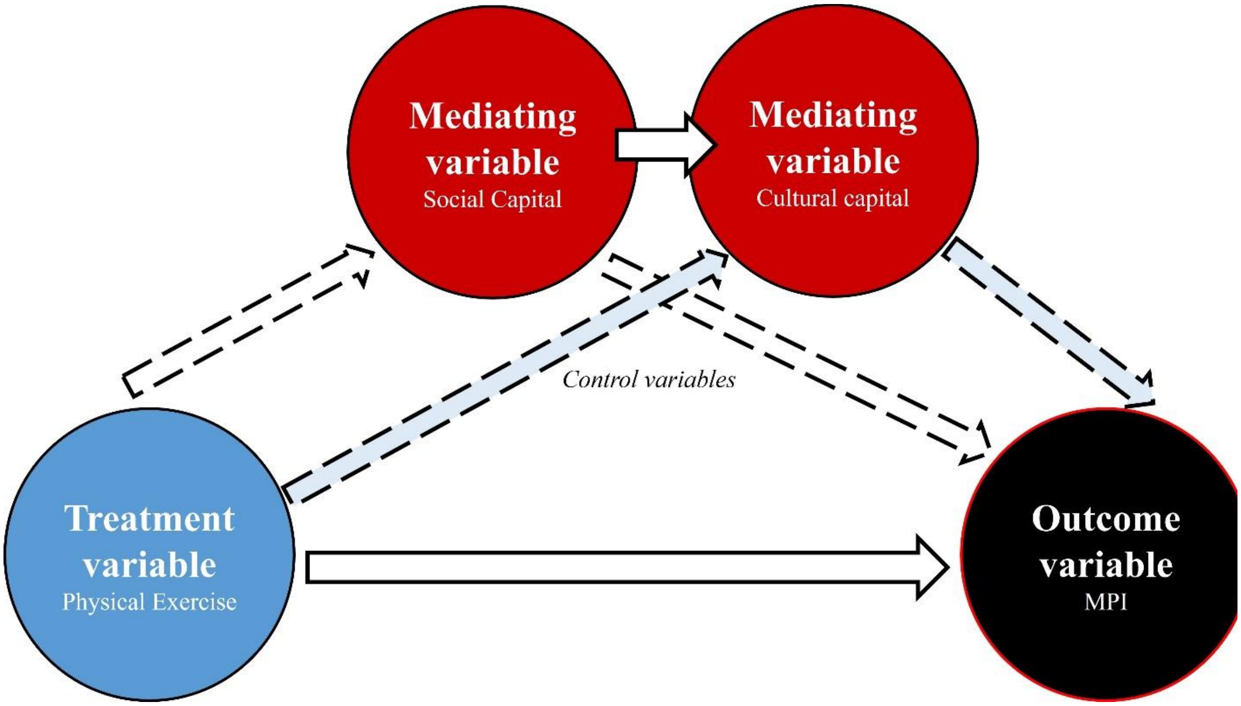

Fig 1b. With connecting mediating variables

- **2.2.4 Heterogeneous Effects Model**: If a different model was used for this analysis, please specify. If one of the models above was used, this should be indicated under the respective subsection.
- **2.2.5 KHD**: Why was this method chosen, and what is its purpose in this paper? If it serves the same purpose as the mediation analysis, why are both methods included?
- **2.2.6 Robustness Checks**: Clearly explain what models were used. The instrumental variables discussed from line 691 need proper description. What were they, and was the exclusion restriction satisfied? In my view, this should be the main model, not OLS.

From this, it becomes evident that the paper attempts to cover too many models, resulting in a lack of focus and coherence in the empirical strategy. It is unclear what the authors are trying to achieve. All model specification explanations currently in the results section should be moved to this new methodology section.

Regarding the results, the authors' recent response leaves much to be desired. It would have been helpful to share the full syntax or screenshots of the results. Ideally, the Stata syntax in your case would be:
sgmediation2 MPI, iv(physical exercise) mv(mediator) cv(control variables)
Why use a method that only allows one mediator at a time when you have two? The estimates from each run do not control for the other mediator and thus are likely biased. This is a flawed approach.

Furthermore, the relatively small mediation proportions (11% and 33%) require clearer explanation. Why is the direct effect (physical exercise → MPI) so much stronger? Additionally, though not critical, the relatively smaller coefficients when cultural capital is used as a mediator need elaboration.

The effect of physical exercise on MPI should be presented robustly. Following this, heterogeneity and moderation (if necessary) analysis could be presented using graphs, as done by Li, Ma, and Gong (2023) in disaggregation analysis. Only after this should the mediation analysis results be introduced—with carefully selected mediators. Social and cultural capital don't seem to fit well, given that this study focuses on **individual** exercise. A well-developed reasoning and theoretical link must be presented in the hypothesis development section.

References

Li, J., Ma, W., & Gong, B. (2023). Market participation and subjective well-being of maize farmers. *Economic Analysis and Policy, 80*, 941-960.

Zhang, J., Mishra, A. K., Zhu, P., & Li, X. (2020). Land rental market and agricultural labor productivity in rural China: A mediation analysis. *World Development, 135*, 105089.
