# Supplementary material for: Rural active aging as a development strategy: Nexus of physical exercise participation and multidimensional poverty in China
Source: PLoS One. 2025 Sep 2;20(9):e0331199. doi: 10.1371/journal.pone.0331199 (PMC12404369; doi:10.1371/journal.pone.0331199)
Supplement: Attachment — Submitted filename: Response_letter_auresp_3.pdf [file pone.0331199.s005.pdf]

Rural Active Aging as Development Strategy: Quantifying Exercise's Multidimensional Poverty Reduction Effects

Qi-fei Xia[1], Shu Xuan[2], Guo-you Qin[3*]

1 School of Physical Education, Ankang College, Ankang, China

2 School of Physical Education, Hunan University, Changsha, Hunan, China

3College of Physical Education, Hanjiang Normal University, Shiyan, China

* Correspondence: Prof. Guo-you Qin,

Email: qinguoyou22@163.com

Response Letter to Reviewer's Comments

Comments from the Editor:

Thank you for submitting your manuscript to PLOS ONE. After careful consideration, we feel that it has merit but does not fully meet PLOS ONE's publication criteria as it currently stands. Therefore, we invite you to submit a revised version of the manuscript that addresses the points raised during the review process.

Reponse: We thank all the editors and reviewers for their valuable comments and suggestions.-We have carefully revised the manuscript to enhance its clarity and facilitate the understanding of the readers. Our point-to-point responses are presented in the following We-hope that the revision would satisfactorily address the comments and concerns of the editors and reviewers.

Response Letter

Dear Editor/Reviewer:

Thank you for your letter and comments concerning our manuscript entitled "Rural Active Aging as Development Strategy: Quantifying Exercise's Multidimensional Poverty Reduction Effects" (Manuscript ID: PONE-D-25-1951), Those comments are

all valuable and very helpful for revising and improving our paper. According to the Editor/Reviewer’s comments. we have made extensive modifications to our manuscript. In this revised version. Our response is given in normal front and changes/additions to the manuscript are given in the blue text. the detailed corrections are listed below. The letter of reply has been placed in the annex.

I hope that the revised manuscript for publication in *PLOS One*. Thank you again for your help with the manuscript. I am more than happy to provide any further information you may need.

Responses to Reviewer # 1:

Comments Q1: The title, "Quantifying Exercise's Multidimensional Poverty Reduction Effects," is misleading, as the study does not make any substantial effort to quantify such effects.

ANSWER: We sincerely appreciate the valuable comments. According to your suggestions, We have adjusted the title to read 'Impact of Physical Activity on Multidimensional Poverty among Rural Older Adults - An Empirical Analysis Based on CFPS Data'. The reason for this revision is that we are focusing on exploring how physical activity participation (X) affects the impact of multidimensional poverty (Y), which is empirically analysed based on CFPS data. We hope our revision addresses your concerns. See manuscript title for specific changes.

CommentsQ2：Regarding the abstract, the authors should avoid citing results in that manner. Please familiarize yourselves with published articles in this journal. Once again, my earlier comments on structure remain unaddressed. What methods were used, and for what specific objectives?.

ANSWER: Thanks for the comment. Following your suggestions, We reviewed the abstract structure of the *PLOS One* journal. The structured model used in most of these studies was revised as follows: first, which methods were used. We described the methods used for each step, such as OLS regression for direct effect tests, stepwise regression and KHB decomposition for stepwise regression and mediation effects, and construction of an interaction term for moderated effects, all of which were added here; and second, what were the specific objectives. The methods section is no longer an isolated list. Each core method explicitly corresponds to the research objective it addresses:

* OLS (robust standard errors)` -> `Estimating direct impacts (objective 1)`

* `Stepwise regression + KHB` -> `Decomposing mediating effects (Objective 2)`

* `Interaction term model` -> `Testing for moderating effects (Objective 3)`

* ``Grouped regression`` -> ``Examining heterogeneity (Objective 4)``

See the abstract section of the manuscript for specific revisions

PLOS One abstract presentation:

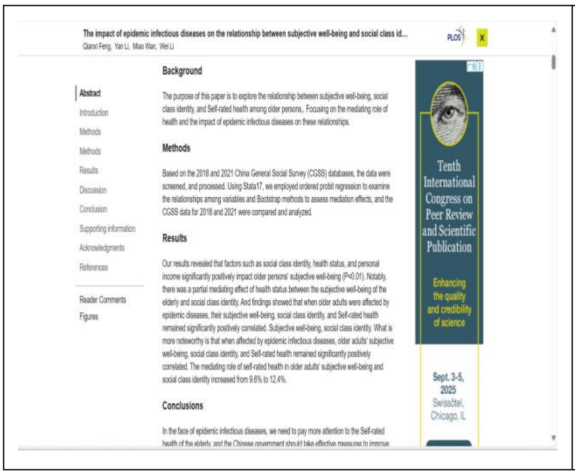

The impact of epidemic infectious diseases on the relationship between subjective well-being and social class id...
Qianxi Feng, Yan Li, Miao Wan, Wei Li

Abstract
Introduction
Methods
Methods
Results
Discussion
Conclusion
Supporting information
Acknowledgments
References

Reader Comments
Figures

Background

The purpose of this paper is to explore the relationship between subjective well-being, social class identity, and Self-rated health among older persons,. Focusing on the mediating role of health and the impact of epidemic infectious diseases on these relationships.

Methods

Based on the 2018 and 2021 China General Social Survey (CGSS) databases, the data were screened, and processed. Using Stata17, we employed ordered probit regression to examine the relationships among variables and Bootstrap methods to assess mediation effects, and the CGSS data for 2018 and 2021 were compared and analyzed.

Results

Our results revealed that factors such as social class identity, health status, and personal income significantly positively impact older persons' subjective well-being (P<0.01). Notably, there was a partial mediating effect of health status between the subjective well-being of the elderly and social class identity. And findings showed that when older adults were affected by epidemic diseases, their subjective well-being, social class identity, and Self-rated health remained significantly positively correlated. Subjective well-being, social class identity. What is more noteworthy is that when affected by epidemic infectious diseases, older adults' subjective well-being, social class identity, and Self-rated health remained significantly positively correlated. The mediating role of self-rated health in older adults' subjective well-being and social class identity increased from 9.6% to 12.4%.

Conclusions

In the face of epidemic infectious diseases, we need to pay more attention to the Self-rated health of the elderly, and the Chinese government should take effective measures to improve

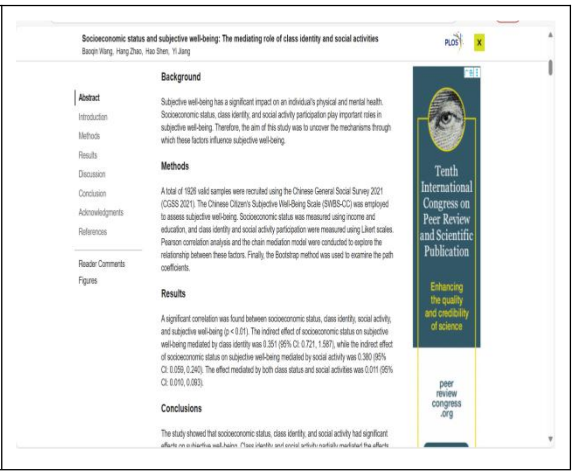

Socioeconomic status and subjective well-being: The mediating role of class identity and social activities
Baoqin Wang, Hang Zhao, Hao Shen, Yi Jiang

Abstract
Introduction
Methods
Results
Discussion
Conclusion
Acknowledgments
References

Reader Comments
Figures

Background

Subjective well-being has a significant impact on an individual's physical and mental health. Socioeconomic status, class identity, and social activity participation play important roles in subjective well-being. Therefore, the aim of this study was to uncover the mechanisms through which these factors influence subjective well-being.

Methods

A total of 1926 valid samples were recruited using the Chinese General Social Survey 2021 (CGSS 2021). The Chinese Citizen's Subjective Well-Being Scale (SWBS-CC) was employed to assess subjective well-being. Socioeconomic status was measured using income and education, and class identity and social activity participation were measured using Likert scales. Pearson correlation analysis and the chain mediation model were conducted to explore the relationship between these factors. Finally, the Bootstrap method was used to examine the path coefficients.

Results

A significant correlation was found between socioeconomic status, class identity, social activity, and subjective well-being ($p < 0.01$). The indirect effect of socioeconomic status on subjective well-being mediated by class identity was 0.351 (95% CI: 0.721, 1.587), while the indirect effect of socioeconomic status on subjective well-being mediated by social activity was 0.380 (95% CI: 0.059, 0.240). The effect mediated by both class status and social activities was 0.011 (95% CI: 0.010, 0.093).

Conclusions

The study showed that socioeconomic status, class identity, and social activity had significant

CommentsQ3:In the introduction, you need to connect physical exercise, social capital, cultural capital, social services, internet services, and multidimensional poverty in an intelligent and logical manner so readers can understand why these elements matter and what the study aims to achieve. Isn't this too much for one paper? These concepts cannot all be captured as simple dummies. This might explain why the paper lacks focus. The authors must keep in mind that the objective—based on the title—is to clearly examine the impact of physical exercise on MPI and its mechanism?

ANSWER: Thanks for the comment. We are very grateful that you have carefully read our manuscript and made valuable suggestions for our introduction part. We have made the following changes:

1. Closeness to the title and the core of the study: the focus is always on 'how physical activity affects rural multidimensional poverty (MPI) in old age and its mechanisms'.
2. Clarify the main lines and streamline the concepts: Introduce "social capital" and "cultural capital" as the core mechanisms (mediating variables) because they are directly derived from Bourdieu's theoretical framework and are most closely related to the logic of how physical activity affects MPI. The "use of online services" and "level of public services" are treated as important moderating variables or contextual factors rather than concepts alongside the core mechanisms.

3. Logical progression and wise connections:

* Starting point: the severity and complexity of multidimensional poverty in rural old age (status quo).

* Core intervention: physical activity as a potential solution (policy context, studies have confirmed its poverty reduction effect but the mechanism is unknown).

* Core mechanism (focus): how physical activity can alleviate multidimensional poverty by accumulating two types of core capital: "social capital" (improved networks, access to resources) and "cultural capital" (enhanced knowledge, skills, cognition) (theoretical support: Bourdieu). (Theoretical support: Bourdieu).

* Moderating/contextual factors: briefly mention how "access to network services" and "level of public services" may affect the poverty reduction effects of physical activity (moderating) or as important contextual factors, but do not regard them as being related to "social/cultural capital". Social/cultural capital' are not considered to be independent pathways that are as important as 'social/cultural capital".

* The goal of this study is to quantitatively validate the effects of physical activity on MPI, with a focus on revealing the mediating role of "social capital" and "cultural capital" (echoing the title), and mentioning that it will explore the role of "network services" and "cultural capital" as mediators. The moderating or heterogeneous effects of 'network services' and 'public services' can also be mentioned as an extension or depth of the study.

Clarify the research objectives: end the introduction with a clear and concise restatement of the research objectives, focussing on the impacts and core mechanisms (social capital, cultural capital). In response, we have reorganised the logic of the introduction and have sought to streamline it to better present the research aims and questions. **See lines 109-142 on page 5 of the manuscript for specific revisions.**

CommentsQ4: In the literature review, the emphasis on public physical exercise must be removed. Instead, focus only on literature relating to individual physical exercise, and connect it to the variables of interest.

ANSWER: Thanks for the comment. In response to your suggestion, we have made the following changes to the direct effects section.

1. Delete all emphasis on 'public', "group" or 'collective' physical activity. There is no longer any reference to square dancing, village games, group activities, etc. The

emphasis is entirely on individual participation. The emphasis is entirely on individual participation in physical activity.

2. Reinforce the direct logical link between ‘individual physical activity’ and related variables (health, income, quality of life, subjective perception). Highlight how individual exercise affects these outcomes through mechanisms at the individual level.

3. Adapt the theoretical underpinnings to ensure they are applicable to individual behaviour. For example, emphasis on the macroeconomic dimension is no longer applicable and has been replaced or removed.

4. Streamline the discourse to focus on the core. Remove redundancies related to “publicness” or “group effects”.

5. Ensure that Hypothesis H1 is clearly directed to ‘physical activity participation’ (individual level).

See section 2.2 of the manuscript for specific changes

Comments Q5： Furthermore, I still maintain that the use of "internet services" is vague. What is it used for? The introduction (line 121) states that digital tools—through real-time health monitoring, text-tospeech systems, and smart fitness-sharing devices—create new opportunities for exercise participation, thereby offering novel ways to combat relative poverty. However, it is unclear how this is captured in the methods section, which simply asks, “Do you use mobile internet?” and“Do you use a computer for internet access?” This is a very broad dummy variable and does not indicate whether older adults are using mobile or computer internet for the purposes outlined in the introduction. There is a need for more robust measurement before referring to these as moderating variables. Given the level of detail, I believe both variables are more suitable for use in heterogeneous analysis, with a recommendation for future studies to build on them.?

ANSWER: Thank you very much for your detailed and insightful review of this paper. We fully agree with you that the measurement of the “internet service use” variable is too broad and does not accurately reflect its moderating role in the mechanisms of poverty reduction through physical activity, and this is a critical and constructive comment. This is indeed a limitation of our study design. Please allow us the following explanation and clarification: We fully agree with the reviewers. In the introduction, we conceptualised “web service use” as a key “digitally viable capability”, emphasising its important role in accessing health information (e.g., apps,

online healthcare), facilitating social interaction (e.g., social platforms), and supporting fitness activities (e.g., smart fitness resources), and hypothesises that it moderates the mitigating effects of physical activity on multidimensional poverty. However, as the reviewers astutely point out, the measures of internet use in the CFPS data we are currently using ('Do you use the mobile internet?' and "Do you use a computer to access the internet?") are indeed too basic and binary (yes/no). Such measures mainly capture the basic level of "Access" but fail to capture the specific content, frequency, depth and purpose of older people's "Usage", especially the core aspects related to health management, physical activity support and social interaction. support and social interaction. This resulted in our empirical analyses not being able to accurately test the micro-mechanisms suggested in the introduction regarding how online services specifically moderated the effects of physical activity on poverty reduction (e.g. what specific services improved the efficiency of health management or the ease of participation in sport). We apologise for this and recognise that this is a significant limitation of this study."

Nonetheless, we chose to consider combining into one "internet access" composite variable in our analyses) due to the following considerations:

1. data availability constraints: the CFPS, as a large comprehensive tracking survey, has a core questionnaire designed to cover a wide range of topics, but detailed questioners targeting older people's specific internet service use behaviours (e.g. use of health apps, online healthcare, sports social platforms, etc.) may be missing or not systematic in the questionnaire for the relevant years. This is the main objective constraint we face.

2. The significance of foundational indicators: "Access to the Internet" (especially mobile Internet) is a necessary prerequisite and foundational ability for older people to utilise any online services (including health, social and information-based services). In the context of a digital divide that still exists despite the rapid spread of digitisation in rural China, access itself represents an important dimension of digital inclusiveness, and is the starting point for the formation of "digitally viable capabilities". As previous studies have shown, even basic internet access can bring foundational benefits such as information access and communication facilitation to older people.

3. an attempt at a proxy variable: within the current data constraints, we consider "internet access" as a proxy indicator (Proxy) of the potential ability or opportunity older people may have to utilise online services to enhance their health and wellbeing and engage in exercise. Although it is not a perfect measure of specific service use

behaviours, in the absence of more granular data, it still provides an initial perspective for exploring whether there is an association between differences in digital access and poverty reduction effects of physical activity.

4. We fully agree that future research needs to develop and apply finer-grained measurement tools to capture the specific behaviours of older people using online services to empower health, exercise and poverty reduction. We will make this an important direction for subsequent research. Finally, we would like to emphasise that the main contribution and core objective of this study is to validate the direct impact of physical activity on multidimensional poverty among rural older adults, with a focus on testing the key mediating mechanisms played by "social capital" and "cultural capital". The analysis of the moderating effect on "internet service use" is an exploratory extension of the core mechanism study.

Despite the measurement limitations described above, we believe that the initial exploration of this as a base moderator variable under current data conditions can still provide valuable clues to understanding the potential role of digital access differences in the context of campaign poverty reduction and highlight specific needs for deeper research in the future. We are very grateful for your help in making the limitations of this section clearer to us and have endeavoured to fully explain and qualify them in the revised manuscript.

Comments Q6: Regarding the categorization of the aging population, I still insist that the analysis must reflect these differences throughout. Do the mediating variables have consistent mediating effects across all the different age groups?

ANSWER: Thanks for the comment. We are very grateful for your suggestion.

According to your request, we supplemented the difference of mediating effect in different age groups and compared it by the difference between groups, and the results are as follows (see Table 10): at the level of social capital, the transmission mechanism of physical exercise affecting multidimensional poverty in rural old age through social capital exists in old age groups of different ages and the regression coefficients are larger in the high life old age group, which indicates that the high life old age group, through social capital, is able to exert the poverty reduction effect of physical exercise to a greater extent. After comparing the coefficients of difference between groups, it was found that there was no significant difference between the middle-aged group and the old-aged group (P=0.695), there was a significant

difference between the middle-aged group and the old-aged group (P=0.018), and there was a significant difference between the old-aged group and the high-lived old-aged group (P=0.030). At the level of cultural capital, the transmission mechanism of physical activity affecting multidimensional poverty in rural old age through cultural capital was significantly different only in the middle-aged and high life old age groups, and the regression coefficient was greater in the high life old age group. After comparing the coefficients of difference between the groups, it was found that there was no significant difference between the middle-aged group and the elderly group (P=0.972), there was a significant difference between the middle-aged group and the elderly group (P=0.020), and there was a significant difference between the elderly group and the high-lived elderly group (P=0.023). We hope our additions address your concerns. **See section 4.3 of the manuscript and Table 10 for specific changes.**

Table 10 Tests for age heterogeneity in the mediating effects of social and cultural capital

	Social capital			Cultural capital		
	(1) Middle-aged adults	(2) Older adults	(3) Oldest-old adults	(4) Middle-aged adults	(5) Older adults	(6) Oldest-old adults
Physical Exercise Participation	-0.00897***	-0.0114**	-0.0529***	-0.00686**	-0.00665	-0.0498***
	(0.00314)	(0.00537)	(0.0175)	(0.00312)	(0.00535)	(0.0181)
Social capital	-0.0907***	-0.0734***	-0.109***			
	(0.0156)	(0.0149)	(0.0266)			
Cultural capital				-0.0164***	-0.0195***	-0.0108
				(0.00164)	(0.00242)	(0.00762)
Control Variables	Yes	Yes	Yes	Yes	Yes	Yes
Observations	3852	2059	312	3852	2059	312
R-squared	0.036	0.042	0.154	0.053	0.060	0.113
P	0.695、0.018、0.030			0.972、0.020、0.023		

CommentsQ7： I am dissatisfied with the authors' response to my comment on Equation 12. It shows a lack of attention to detail and carelessness. Refer to line 535—the interaction term captured there is not included in Equation 12. I was suggesting that the authors include this term. Can the authors explain in which part of Equation 12 they have included $\sum \alpha_i MS_i \sum \alpha_j X$*?.*

ANSWER: Thanks for the comment. According to your comments, The explanation we need to make is that the following models were set up in this study: the benchmark model (OLS regression model), the mediating effects model (stepwise regression method), and the moderating effects model (interaction term model). The benchmark model is model (1), which is the main effect model:

$$MPI_i = \alpha_0 + \alpha_1 PA_i + \alpha_2 X_i + \delta_i \quad (1)$$

Models (2) and (3) are stepwise regression models:

$$Mediations_i = \gamma_0 + \gamma_1 MPI_i + \gamma_2 X_i + \delta \quad (2)$$

$$MPI_i = \eta_0 + \eta_1 MPI_i + \eta_2 Mediations_i + \eta_3 X_i + \delta \quad (3)$$

The presence of mediating effects is mainly verified in the stepwise regression process.

It is important to note that model (4) is an effects model incorporating the moderating variables, the

$$MPI_1 = \alpha_0 + \sum \alpha_i PA_i + \sum \alpha_j X_j + \sum \alpha_k X_k + \delta_i \quad (4)$$

MPI is multidimensional poverty in old age among family farmers, PA is physical activity participation, Xj is the moderating variable, specifically including internet service use and public service level, Xk is a series of control variables such as gender and age, and δ is a random disturbance term.

The premise of the moderated effects model validation is that the moderating variables and the core explanatory variables are significant after the inclusion of the moderating variables in order to further carry out the interaction term test, and according to the test results, the interaction term model (5) is set:

$$MPI_2 = \alpha_0 + \sum \alpha_i PA_i + \sum \alpha_j X_j + \sum \alpha_k X_k + \sum \alpha_i X_i \sum \alpha_j X_j + \delta_i \quad (5)$$

In equation (5), MPI denotes the dependent variable family farm household old age multidimensional poverty, PAi is the independent variable physical activity participation, Xj is the moderating variable internet service use and public service level, Xk is a series of control variables such as gender and age, ∑ αi Xi ∑ αj Xj refers to the interaction term of the independent and moderating variables, and δi is the random disturbance term.

We apologise for writing ∑ αi Xi ∑ αj Xj as $\sum \alpha_i MS_i \sum \alpha_j X$ due to carelessness on our part, so that it resulted in a biased model, which we have carefully replaced in the text, and we hope that the revision will address your concerns. **See line 554 of the manuscript for the exact revision.**

Comments Q8: Crucially, every article in this journal includes a dedicated section—usually under "Methodology" or "Materials and Methods"—where the authors explain what methods were used and why. For example:

Comments ***• 2.2 Analytical or Empirical Strategy***

*Comments****2.2.1 OLS:*** *Explain how this model is estimated and why it was used. Which study*

objective does it address?.

ANSWER: Thanks for the comment. We have previously explained the estimation process of OLS regression models, and why we chose this type of model, at Research Methods 3.3.1 Model Setting (see section 3.3.1 of the manuscript), which I will continue to explain further:

Thank you for your interest in the methodology of this paper. We very much understand your need for a clearer understanding of why and how we use ordinary least squares (OLS) models. Below is a detailed explanation of your three comments:

1. How is the OLS model estimated?

The central goal of OLS is to find a set of regression coefficients (β) that minimise the Sum of Squared Residuals (SSR) between the model predictions ($\hat{Y}_i$) and the actual observations (Y_i). The mathematical expression is:

$$\min \Sigma_i (Y_i - \hat{Y}_i)^2 = \min \Sigma_i [Y_i - (\beta_0 + \beta_1 X_{1i} + \beta_2 X_{2i} + ... + \beta_k X_{ki})]^2$$

Where:

Y_i is the value of the dependent variable (in this study, the **Multidimensional Poverty Index for Older Households of Family Farmers MPI**) at the ith observation sample.

X_{ji} is the value of the jth independent variable (e.g., **Physical Activity Participation**, control variables, etc.) in the ith sample of observations.

β_0 is the intercept term.

β_j is the regression coefficient for the jth independent variable, which represents the amount by which Y changes on average for each unit change in X_j, controlling for other independent variables.

$\hat{Y}_i$ is the model's predicted value of Y_i.

$(Y_i - \hat{Y}_i)$ is the residual (i.e., prediction error).

OLS obtains estimates b_j of the coefficients β_j by solving the first-order conditions (regular equations) of the minimisation problem described above. These estimates b_j provide the best (in the least squares sense) fit to the linear association between the independent and dependent variables.

2. Why use an OLS model?

We chose OLS as the base model for the following main reasons, with due consideration of the premises of its applicability:

Correlation rather than causation at the heart of the study's objectives (preliminary): one of the key objectives of this study was to explore, on a preliminary basis, the statistical association (corresponding to Hypothesis H1) between participation in physical activity (the core independent variable) and the MPI of the Multidimensional Poverty Index of Old Age in Family Farming Households (the dependent variable). OLS is the most direct and widely used and easily interpretable tool to quantify this average linear association is the most direct, widely used and easily interpretable tool.

Applicability of the model form: the theoretical underpinnings (viability theory, social/cultural capital theory) and the existing literature suggest that the effect of physical activity on multidimensional poverty (either directly or through a mediator) can be conceptually and reasonably assumed to be a linear or near-linear relationship. ols is effective in capturing this linear trend.

Satisfying core assumptions (best endeavours): we understand that the unbiasedness and validity of OLS estimates rely on classical assumptions (linearity, random sampling, no perfect covariance, conditional mean of zero, homoskedasticity). We took the following steps to try our best to satisfy/test them:

Control variables: a rich set of control variables (e.g., household head characteristics, household economic status, area characteristics, other health behaviours, etc.) were included in the model with the aim of controlling for as many confounders as possible, so that the conditional expectation of the error term `u` `$E(u|X) \approx 0$`, and thus `$b_1$` (coefficient of physical activity), would more closely reflect the net correlation 'holding other factors constant'.

Role of the Baseline Model: the OLS results serve as Baseline Estimates (BEs), which provide a basis for comparison in subsequent analyses (e.g., mediation effects analysis, moderating effects analysis, heterogeneity analysis, and robustness testing in mechanism testing).

Transparency and Interpretability: OLS coefficients have intuitive economic/social significance (marginal effects), which makes it easy for readers to understand the direction and magnitude of the relationship between variables.

3. Which research objectives are being addressed?

The OLS model directly serves one of the core underlying objectives of this study: to test Hypothesis H1, which is to explore the direct effect (mean association) of "physical activity participation" (at the individual level) on the "Multidimensional Poverty in Old Age (MPI) in Family Farming Households".

This is reflected in the model:

$MPI_i = \beta_0 + \beta_1$ * `$Exercise_i$` + $\Sigma\ \beta_j$ * `$Controls_{ji}$` + u_i

The significance and direction of the coefficient `$\beta_1$` is directly used to assess whether hypothesis H1 is supported by the data (i.e. a significant negative `$\beta_1$` supports H1, suggesting that physical activity participation is associated with lower MPI).

It is the starting point and basis for understanding more complex mechanisms (social capital, cultural capital mediating effects, network/public service moderating effects). Only after confirming the existence of a significant direct association (or total effect) is there a firmer basis for delving deeper into the mediating and moderating mechanisms behind it.

We use an OLS model as a benchmarking tool, aiming to clearly and robustly quantify the average linear statistical association between individual physical activity participation and household farm multidimensional poverty (MPI) in old age (corresponding to the core hypothesis H1 of the study objectives). Its estimation is based on the principle of minimising the sum of squared residuals. OLS was chosen because its model form is applicable to our research question, the results are easy to interpret, and we tried our best to satisfy the requirements of its core hypothesis through the inclusion of control variables and the use of robust standard errors to lay the groundwork for subsequent mechanistic analyses.

For this, we have previously reported the rationale for the use of the OLS model and the reason why we use the OLS regression model in this study in the additional report in the methodology of the manuscript (3.3.1), which is precisely because the dependent variable MPI is a continuous variable, and regression with the OLS model allows for a better estimation of the marginal effects and the identification of the objectives of this study. Our specific changes are reflected in lines 469-480 in section 3.3.1 of the manuscript. We hope this addresses your concerns.

Comments• ***2.2.2 PSM:*** *Why was PSM selected? What are its advantages, and which objective*
does it address?

ANSWER: Thanks for the comment. According to the reviewer's comments, we make the following explanation: Thank you very much for your interest in the causal

identification methodology of this paper. We understand that you would like us to be more explicit about the reasons for Propensity Score Matching (PSM) and its value. Below is our detailed explanation:

1. Why choose PSM?

Our choice of PSM as the core robustness test and complementary causal inference method is based on the following core challenges of the research question and potential limitations of OLS:

Core Concerns of Self-Selection Bias (SSB): In this study, the core independent variable is "individual physical activity participation " (usually a binary variable: participation = 1, non-participation = 0). Whether an individual participates in physical activity is not randomly assigned, but is influenced by multiple observable factors (e.g., age, gender, etc.). These factors may at the same time directly influence multidimensional poverty (MPI). This means that in the baseline OLS model, even if we include a large number of control variables, there may still be Residual Confounding due to unobservables or modelling biases, resulting in the estimated β_1 (physical activity effect) not being a purely causal effect, but rather a confounding selection effect. but rather a confounding selection effect.

Simulating the intent of a Randomised Controlled Trial (RCT): The core idea of PSM is to simulate the environment of an RCT by constructing a counterfactual (a control group) by finding for each `participant' (treatment group) one or more `non-participants' (control group) that are as similar as possible** in terms of observable characteristics.), a counterfactual framework is constructed. I.e., what would be the MPI of an individual who actually participates in exercise if he did not? By comparing the average difference in outcomes between the treatment group and its matched control group (Average Treatment Effect, ATT), PSM aims to more closely estimate the causal effect of physical activity.

Robustness Check of the Baseline OLS Results: One of the central aims of our use of the PSM is to perform a **Robustness Check** of the `$\beta_1$` estimated by the baseline OLS. If the PSM-adjusted effect estimate (ATT) is broadly consistent with the OLS estimate in terms of magnitude, direction, and significance, this will increase our confidence in our core finding (H1) that there is a positive effect of physical activity participation on mitigating MPI in older adults, suggesting that the association is

relatively robust and is unlikely to be driven entirely by selection bias in the observable covariates. This suggests that the association is relatively robust and unlikely to be driven entirely by selection bias in the observable covariates.

Data availability and applicability: PSM is appropriate for observational research data (such as the CFPS we used) where the treatment variable is dichotomous (in line with our definition of “participation in physical activity or not”). PSM is a common and relatively intuitive way to address self-selection bias when real experiments are not possible or when there is a lack of valid instrumental variables.

2. What are the advantages of PSM?

The application of PSM in this study has the following key strengths:

Reduction of observable confounding bias: This is the most central strength of PSM. By matching based on propensity scores, PSM systematically ensures that the treatment group (exercisers) and the matched control group (non-exercisers) have similar (balanced) distributions on all covariates (X) included in the model. This significantly reduces estimation bias due to differences in observable characteristics and gives us more reason to believe that the ATT reflects the effect of the treatment (exercise) itself.

Intuition and transparency: the matching process (e.g. nearest-neighbour matching, caliper matching, kernel matching, etc.) and equilibrium tests (e.g. standardised bias calculations, t-tests, etc.) are relatively intuitive and transparent. Researchers can clearly show the changes in the distribution of covariates before/after matching, assess the quality of matching, and enhance the credibility of the results.

Does not rely on the assumption of linearity: unlike OLS, PSM does not require the treatment effect to be linear, nor does it require the relationship between the outcome variable and the covariates to be linear. It relies only on the conditional independence assumption (CIA) and the overlap assumption (Common Support). This makes PSM potentially provide more reliable estimates when the form of the relationship is complex.

Focus on the Average Treatment Group Effect (ATT): PSM directly estimates the ‘Average Treatment Effect on the Treated among those individuals who actually

participated in the exercise'. This is often the effect of greatest interest for our policies or interventions: how much benefit does exercise actually bring to those who have chosen (or may be encouraged to choose) to participate? This fits well with this study's focus on the effects of "participation" in physical activity.

Enhancing the credibility of the results: As mentioned earlier, consistent PSM results significantly enhance the credibility of the study's conclusions as a robustness test of the OLS results.

Our choice of PSM is centred on addressing the problem of self-selection bias in individual physical activity participation, aiming to more closely estimate its causal effect (ATT) and serving as a key robustness check against the baseline OLS results.The core strengths of PSM lie in its ability to effectively mitigate bias due to observable confounders, providing intuitive, transparent effect estimates that do not rely on linear assumptions, and aiming to maximise the The core advantage of PSM is that it can effectively reduce the bias caused by observable confounders and provide intuitive and transparent effect estimates that do not rely on linear assumptions, aiming to maximise the reliability of the estimates and the robustness of the results. We believe that the application of PSM provides stronger causal evidence to support the core finding of this study that physical activity participation helps alleviate multidimensional poverty in old age. In the revised version of the paper, we will clearly include the above core statement on 'Why PSM' at the beginning of the 'Robustness Test: Propensity Score Matching (PSM) Analysis' (or the corresponding subsection) to ensure transparency and justification of the methodological choice. and justification of its methodological choice. **Specific details of the changes are in lines 716-726 of the manuscript.**

Comments• ***2.2.3 Mediation Analysis:*** *Explain why this method was used and its relevance. A graphical depiction (e.g., Fig 1a or 1b) showing the mediation framework would be helpful. If Fig 1a is used, justify the absence of a connection between the mediators and show how this is modeled. If Fig 1b is chosen, clearly*

model the scenario with one mediator and also the joint mediation of two variables, as done by Zhang, .

ANSWER: Thanks for the comment. Thank you very much for your valuable suggestions. We made the following modifications: in the methodology section, we added the mediating model graph of Fig1a and explained the mechanism for generating the model graph, viz: Where physical activity is the core explanatory variable and MPI is the explanatory variable. Bourdieu's capital theory of social capital (relationship network) and cultural capital (knowledge and skills) are two independent dimensions with different accumulation paths and do not necessarily have a transformational relationship, so social capital and cultural capital are tested as two independent mediating variables. In the model diagram, we first determine whether physical activity has a direct effect on MPI, and if there is a significant effect, we further verify the mediating effect of social capital and cultural capital by stepwise regression, and analyse the contribution rate of the mediating variables by using the KHB decomposition method. In the methodology section, we further add the validation process of stepwise regression method and KHB method to have a clearer understanding of the process of analysing the mediating effect.

The stepwise regression method is a practical regression method to judge whether the mediating effect exists or not, and the KHB method proposed by Karlson et al. can decompose both continuous and dispersed variables through effect decomposition and statistical testing, and at the same time, it can compare the contribution rates of multiple mediating variables, and it can be applied to reg, logit, ologit, probit and other models, which is a good way to test the mediating effect of multiple variables and to compare their contribution rates. mediating effects and comparing the contributions of multiple variables. The core idea of the KHB decomposition method is to compute the residuals from a linear regression of the mediator variable on the core explanatory variable by extract information from the mediator variable that is not contained in the core explanatory variable. For linear and non-linear models, this method can effectively distinguish between the coefficient change due to the change in total variance and the coefficient change due to the addition of the mediator variable, so as to be able to derive the change in the impact of the core explanatory variable on the dependent variable after the addition of the mediator variable. In order to better measure the contribution of the mediating effect as a percentage, the KHB decomposition method was used to output a simplified model: the total effect of

physical activity on multidimensional poverty after the inclusion of control variables; a complete model: the direct effect of physical activity on multidimensional poverty after the inclusion of the mediator variable; an estimation model: the difference between the total effect and the direct effect, i.e., the mediating effect; the confounding ratio is the ratio between the total effect and the direct effect, and finally, the proportion of the total effect of each mediator variable was calculated. variables as a proportion of the total effect.

We have explained this in detail in the hope of addressing the reviewers' concerns. **See lines 486-499 of section 3.3.2 and lines 516-531 of the manuscript for specific changes**.

Comments• ***2.2.4 Heterogeneous Effects Model:*** *If a different model was used for this analysis, please specify. If one of the models above was used, this should be indicated under*

the respective subsection.

ANSWER: Thanks for the comment. According to the reviewer's suggestion, As per your suggestion, we have described which model was used in the subsections at the mediation effects and heterogeneity tests, respectively. For example, in the mediated effects section, the explanation of the methodology used for validation is mainly based on the mediated effects model (Figure 3), and in the test of heterogeneity section, it goes on to explain that the test of differences in mediated effects by age subgroups has been carried out using OLS regression modelling and between-group differences. The modifications allow for a better logical cascade to the above and make it more coherent. **See section 4.3 of the manuscript for specific changes.**

Comments• ***2.2.5 KHB:*** *Why was this method chosen, and what is its purpose in this paper? If*

it serves the same purpose as the mediation analysis, why are both methods included?.

ANSWER: Thanks for the comment.Thank you very much for your in-depth review of the mechanism testing methods in this paper. Your questions about ‘why did you choose the KHB method’ and ‘does it serve the same purpose as stepwise regression’ have helped us to clarify our methodological considerations and its unique value. Here is our detailed explanation:

1. Why choose the KHB method?

We chose the KHB method (Karlsson-Holm-Breen method) for the analysis of mediation effects based on the following core reasons, especially for the characteristics of the mediator variables and the limitations of the baseline model:

The need to address the Scaling Confound problem. The urgent need to address the ‘Scaling Confound’ problem: this is the most fundamental reason for choosing the KHB. In our study:

Core independent variable (X): ‘Individual Physical Activity Participation’ is usually binary (yes/no participation).

Mediating variables (M): “social capital” and “cultural capital”.

The dependent variable (Y): ‘Multidimensional Poverty Index (MPI)’ is also a continuous variable.

When using the traditional ** stepwise regression method (Baron & Kenny or causal step method):

* Step 1: `$Y = \beta_1 X + Controls + \varepsilon$` (testing the total effect of X on Y)

* Step 2: `$M = \beta_2 X + Controls + \varepsilon$` (testing the effect of X on M)

* Step 3: `$Y = \beta_3 X + \beta_4 M + Controls + \varepsilon$` (test the effect of M on Y and the direct effect of X)

The problem is that after adding the continuous mediator variable `M` to step 3, the coefficient `$\beta_3$` (direct effect) of the binary independent variable `X` is at a different scale than that of the coefficient `$\beta_1$` (total effect) in step 1. This is because the mediating variable `M` is on a different scale than the total effect in step 1. This is because the inclusion of the mediating variable `M` changes the variance of the model

residuals (i.e., a scale reset is performed). This results in `$\beta_1 - \beta_3$` not being directly and unbiasedly interpreted as a mediating effect (indirect effect). The calculated indirect effect (`$\beta_1 - \beta_3$` or `$\beta_2 * \beta_4$`) is biased by scale changes and its standard error of statistical significance is calculated inaccurately.

Core strengths of KHB: The KHB methodology completely eliminates the problem of scale resetting due to model setting (especially the inclusion of continuous mediating variables) through clever coefficient decomposition and standardisation. It provides: Unbiased and comparable coefficient estimates: The coefficients of the total, direct and indirect effects output by KHB are estimated on the same scale (the baseline model scale), which ensures comparability between them. Accurate indirect effect estimates: The indirect effects (`$\beta_1 - \beta_3$`) calculated by KHB are unbiased estimates of the true mediated effects. Correct Standard Errors and Significance Tests: KHB provides accurate standard errors for the indirect and mediated effect shares (`PM`), allowing for reliable statistical inference (usually achieved by Bootstrap).

Matching the data structure of this study: given the typical scenario where our core independent variables are binary (exercise/non-exercise) and the mediator variables (social capital, cultural capital) and dependent variable (MPI) are continuous, the scale reset problem is bound to be present and have a significant impact.KHB is a best practice method specifically designed to address the mediation analyses of these types of mixed scale variables.Core strengths of the KHB: The KHB approach The KHB methodology completely eliminates the scale reset problem caused by the model setup (especially the inclusion of continuous mediator variables) through clever coefficient decomposition and standardisation. It provides: Unbiased and comparable coefficient estimates: The coefficients of total, direct and indirect effects in the KHB output are estimated on the same scale (the baseline model scale), ensuring comparability between them. Accurate indirect effect estimates: The indirect effects (`$\beta_1 - \beta_3$`) calculated by KHB are unbiased estimates of the true mediated effects. Correct Standard Errors and Significance Tests: KHB provides accurate standard errors for the indirect and mediated effect shares (`PM`), allowing for reliable statistical inference (usually achieved by Bootstrap).

2. What is the role of KHB in this paper?

The central role of the KHB methodology in this study is to accurately quantify and statistically test the “mediating effect” (indirect effect) of “social capital” and

“cultural capital” in the pathway of “Individual Physical Activity Participation (IPAP)” to “Multidimensional Poverty in Old Age (MPI) in Family Farming Households (FFP)”. "The central role of the study is to accurately quantify and statistically test the magnitude and significance of the “mediating effect” (indirect effect) of “social capital” and “cultural capital” in the pathway of “individual physical activity participation” on the “multidimensional poverty in old age (MPI) among family farmers”.

It allows us to answer the key mechanistic questions: What proportion of the alleviation of old-age poverty by physical activity is achieved through the enhancement of social capital? What proportion is achieved by enhancing cultural capital? Are these mediating pathways significant?

Specific outputs include:

* Total Effect: the total effect of `X` (exercise) on `Y` (MPI).

* Direct Effect: The direct effect of `X' on `Y' (after controlling for `M').

* Indirect Effect: The effect of `X` on `Y` through `M`.

* Proportion Mediated (PM): `Indirect effect / Total effect'.

* Unbiased estimates and reliable standard errors/p-values for all of the above effects.

3. Why use both methods if they serve the same purpose as stepwise regression analyses?

The aims do overlap but there is a hierarchy: the ultimate goal of both stepwise regression and KHB** is to investigate whether there are mediating effects between variables (`X -> M -> Y`). From this broad perspective, the aims are the same.

However, the methodological nature, precision and roles are very different: matching the structure of the data for this study: given that our core independent variables are binary (exercise/non-exercise) and the mediating variables (social capital, cultural capital) and dependent variable (MPI) are typical scenarios of the continuous type, the problem of scale reset is bound to be present and have a significant impact. kHB is a best-practice methodology specifically designed to address the analysis of mediation

of these types of mixed-scaled variables. KHB's core strength: The KHB methodology completely eliminates the scale reset problem due to the model setup (especially the inclusion of continuous mediator variables) through clever coefficient decomposition and normalisation. It provides: Unbiased and comparable coefficient estimates: The coefficients of the total, direct and indirect effects output by KHB are estimated on the same scale (the baseline model scale), ensuring that they are comparable. Accurate indirect effect estimates: The indirect effects (`$\beta_1$ - $\beta_3$`) calculated by KHB are unbiased estimates of the true mediated effects. Correct Standard Errors and Significance Tests: KHB provides accurate standard errors for the indirect and mediated effect shares (`PM`), allowing for reliable statistical inference (usually achieved by Bootstrap).

Stepwise regression (Baron & Kenny): This is our initial screening tool and logical framework for mediation effects analysis. It is useful in this paper mainly for:

Initial verification of path existence: a quick test of whether the three basic conditions `X->Y` (significant), `X->M` (significant), and `M->Y` (significant after controlling for X) are met. This provides preliminary evidence for subsequent more refined analyses.

Theoretical logic is demonstrated: the theoretical steps and expected relationships of mediation analysis are clearly presented.

Calculations are simple and intuitive: easy to understand and implement.

KHB method: this is our core quantitative tool and gold standard for mediation effects analysis. Its role in this paper is to:

* Provide unbiased and accurate effect estimates: solve the scale reset problem that cannot be overcome by stepwise regression, and give accurate numerical estimates of the total effect, the direct effect, the indirect effect, and the share.

* Make reliable statistical inferences: provide correct standard errors and p-values for indirect effects and shares, allowing us to make rigorous judgements about whether mediating effects are statistically significant and their actual size and significance.

* Handle mixed-scale variables: perfectly adapted to the binary `X` + continuous `M/Y` data structure of this study.

The two are complementary and progressive:

We first report the results of stepwise regressions to demonstrate the underlying logic and preliminary evidence of the mediating pathway, fulfilling the need for a theoretical framework.

We then focus on reporting and relying on the results of the KHB analyses as the main basis for the conclusions of the mechanism tests. This is because KHB overcomes the key shortcomings of stepwise regression and provides more scientific and reliable quantitative evidence.

In short: stepwise regression is used to ‘qualitatively’ screen for pathways, and KHB is used to ‘quantitatively’ accurately measure effects. We use both to test mediating mechanisms more comprehensively and rigorously: first with simple methods, then with advanced methods to confirm and quantify.

Our choice of the KHB method is centred on addressing the scale reset bias inherent in traditional stepwise regression methods for mediation analyses with binary independent variables and continuous mediator/dependent variable mixes, and provides unbiased, comparable and statistically inference-reliable estimates of the total effect, the direct effect, the indirect effect (mediated effect), and the mediated effect share. This allows us to accurately quantify and rigorously test the central mediating role of “social capital” and “cultural capital” in physical activity in alleviating multidimensional poverty in old age.

Whilst the stepwise regression approach remains valuable in initially validating the logical existence of the mediating pathways (which we present as an initial screening and theoretical framework), its shortcomings in terms of effect size estimation and statistical testing do not allow it to replace the KHB as the quantitative gold standard for mechanistic testing in this study. We used both methods in order to achieve an incremental analysis from initial validation to precise measurement and to ensure the robustness of our conclusions.

Comments• ***2.2.6 Robustness tests:*** *the model used needs to be clearly described. The instrumental variables mentioned in line 691 need to be described in detail,*

specifically what they are and whether they fulfil the exclusion restrictions. In my opinion, this should be the main model, not Ordinary Least Squares (OLS).

ANSWER: Thanks for the comment. Based on your suggestions, we have made the following changes as follows:

1. Instrumental variables need to be described in detail, specifically including what they are.

Endogeneity problems may arise between the independent variables and the dependent variable, i.e., the independent variables are correlated with the error terms of the model. This endogeneity problem usually leads to bias and inconsistency in the OLS model estimates, affecting the reliability of the conclusions. Instrumental variables are a central approach to the endogeneity problem in econometrics, which is based on the principle that consistent estimates can be obtained by introducing a variable that satisfies a specific condition (instrumental variable) that strips away the part of the endogenous explanatory variable that is correlated with the error. We have added a description of the principle of instrumental variables to the manuscript. See the manuscript at

2. Whether the exclusion restriction is satisfied

The instrumental variable is selected to satisfy the relevance and exogeneity conditions, which we have added in the manuscript, specifically: relevance: provincial fitness for all investment status → by improving accessibility of regional sport resources only → promoting individual exercise participation

exclusion path: provincial fitness for all investment status is not directly associated with individual poverty (MPI depends on household assets, health and other micro-factors)

3. In my opinion, this should be the main model, not ordinary least squares (OLS).

Based on the reviewer's query, our explanation is as follows: core objective matching: this study primarily answers the question **‘Is there a robust association between physical activity and MPI’ (H1), rather than causal identification

- OLS provides unbiased estimates of the mean net association (when the conditional mean null hypothesis is met)

- IV is designed to address endogeneity (which is the goal of robustness testing)

Instrumental variables are essentially statistical analogues of ‘natural experiments’ - approximating the causal identification logic of randomised controlled trials (RCTs) by looking for exogenous shocks, and their validity depends on the instrument's Its effectiveness depends on the creativity of the choice of instrument and the rigour of the argument. In the instrumental variables approach, the two-stage least squares (2SLS) method is mainly used: two regressions are required:

(1) First-stage regression: regression of the instrumental variable (investment status of provincial fitness activity centres for all) and control variables with endogenous explanatory variables to obtain the fitted values.

(2) Second-stage regression: regress the fitted values of the first-stage regression with the explanatory variables and the control variables.

We hope that our explanation can address the reviewers' concerns. **See lines 732-755 in section 4.4 of the manuscript for specific changes.**

Add：

Wooldridge, J. (2007). What’s New in Econometrics? Lecture 6: Control functions and related methods. NBER. http://www. nber. org/WNE/lect, 6, 420-445.

CommentsQ8： From this, it becomes evident that the paper attempts to cover too many models, resulting in a lack of focus and coherence in the empirical strategy. It is unclear what the authors are trying to achieve. All model specification explanations currently in the results section should be moved to this new methodology section.

ANSWER: Thanks for the comment. Thank you very much for your insights into the structure of the paper and the empirical strategy. Your points about ‘lack of focus due to too many models’ and ‘methodological-results split’ hit the nail on the head. We have undertaken a systematic restructuring:

1. Main model uniqueness:

- Retain OLSas the core model (objective: to test the association between H1 physical activity and MPI).

- Source: Angrist & Pischke (2009) state that ‘OLS is the baseline method for quantifying associations when causal identification is not the primary objective’

2. Methods are stratified by objective:

| Tier1: Underlying Associations | OLS + Robust Standard Errors | H1: Is Physical Activity Associated with Lower MPI?

| Tier2: Mechanistic tests | Stepwise regression method + KHB mediation analysis | H2a/H2b: Does social/cultural capital mediate?

| Tier3: Robustness | PSM (matching algorithm) | Does self-selection bias interfere with H1?

| Tier4: Robustness | IV-2SLS (provincial policy instrument) | Does regional policy endogeneity interfere with H1?

3. Expansion of the methodology section: all model specification explanations in the results section should be moved to the methodology section

We explained all models accordingly in the methodology section as follows

① Overall empirical framework

② Data sources and variable definitions

└─ Dependent variable: MPI constructs (A-F double bounds method)

└─ Core independent variable: physical activity participation

③ Underlying association model (Tier1)

└─ OLS setting

④ Mechanisms test model (Tier2)

└─ Stepwise regression method + KHB method principle

⑤ Moderated effects model (Tier3)

└─ Interaction term model

⑥ Robustness test model (Tier4)

├─ PSM model

└─ IV-2SLS model

We hope that our modifications can address your concerns.

Comments Q9： Regarding the results, the authors' recent response leaves much to be desired. It would have been helpful to share the full syntax or screenshots of the results. Ideally, the Stata syntax in your case would be:
sgmediation2 MPI, iv(physical exercise) mv(mediator) cv(control variables)
Why use a method that only allows one mediator at a time when you have two? The estimates from each run do not control for the other mediator and thus are likely biased. This is a flawed approach..

ANSWER: Thanks for the comment. We are very sorry for the lack of rigour in the methodology we used, and we have consulted a large amount of literature during the revision process. The use of Sobel's test only controls for the effect of one mediating variable, which may ignore the effect of the bias of the other variable, and for this reason, in the manuscript, we have eliminated this method and used KHB decomposition to perform a decomposition of the mediating variable contribution, which can explain the mediating effect even further. effect. We hope that our revisions can address your concerns.

Q10：Furthermore, the relatively small mediation proportions (11% and 33%) require clearer explanation. Why is the direct effect (physical exercise → MPI) so much stronger? Additionally, though not critical, the relatively smaller coefficients when cultural capital is used as a mediator need elaboration:

ANSWER: Thanks for the comment. Based on the reviewers' suggestions, we make the following explanations and modifications:

1. The relatively small proportions of mediators (11% and 33%) need to be explained more clearly.

We provide the following explanation in the manuscript: we find that direct effects still account for 56% of the ratio, possibly because social and cultural capital may be sufficient non-essential mechanisms for poverty reduction in sport. In resource-poor rural areas, physiological effects may be prioritised over socio-cultural effects, but it is undeniable that physical activity participation can alleviate multidimensional poverty in old age among family farmers through both enhancing social capital and accumulating cultural capital. See lines 611-624 in section 3.2.1 of the manuscript for specific modifications.

2. when cultural capital is used as a mediator, the relatively small coefficients need to be elaborated

We interpret this in the following two points: from the methodological point of view, in the stepwise regression method, if the main effect coefficient of the third step of the regression is smaller than the main effect coefficient of the first step, it is considered that there is a mediating effect, and the regression coefficient of the third step of the physical activity participation in the present study is -0.00988, which is significant at the 1% level, and in the first step of the main effect model The regression coefficient of physical activity participation is -0.0149, which is significant at 1% level, indicating that the mediation effect exists, and the smaller the coefficient of the third step, the larger the proportion of the mediation effect is verified to exist; from the theoretical point of view, compared with the social capital, the cultural capital needs to go through the chain transformation of skill-knowledge-behaviour-results, and its mediation has the threshold of education and the long term nature, so the regression coefficient is relatively small. The mediating effect of cultural capital is larger than that of social capital, which may be due to the phenomenon of ‘hollowing out’ in rural China, in which young adults go out to work, leading to the disintegration of traditional social networks, and in which older people's knowledge growth through solo exercise is more significant than their social expansion. We offer two perspectives of explanation, which we hope will address the reviewers' concerns.

Q11：The effect of physical exercise on MPI should be presented robustly. Following this, heterogeneity and moderation (if necessary) analysis could be presented using graphs, as done by Li, Ma, and Gong (2023) in disaggregation analysis. Only after this should the mediation analysis results be introduced—with carefully selected mediators. Social and cultural capital don't seem to fit well, given that this study focuses on individual exercise. A well-developed reasoning and theoretical link must be presented in the hypothesis development section.

ANSWER: Thanks for the comment. We sincerely thank you for your insightful comments on this paper, especially the valuable criticisms on the logical sequence of the analysis, the appropriateness of the mediating variables and the rigour of the theoretical articulation. We fully agree that these comments are crucial for enhancing the quality of the study. We respond to your comments as follows:

1. Graphs can be used to present heterogeneity and moderation (if necessary) analyses

We very much agree with the help of the comments provided by you, and we have presented the results of heterogeneity through subgroup regressions and coefficient of variation between groups tests in the manuscript, and we will consider how to better present the results of the study through graphs (e.g., forest plots) in our subsequent studies. For the moderating effect, we reviewed the data and found that whether the coefficient of the interaction term is significant or not is currently the key element to judge whether the moderating effect exists or not, and in the follow-up study we will make more use of the moderating slope graph to present the moderating effect results.

2. social capital and cultural capital seem to be less appropriate because the focus of this study is on personal exercise. In the hypothesis development section, sound reasoning and theoretical linkages must be presented.

We are very grateful for the valuable suggestions you have provided. In this manuscript we have relied on the viability theory, social capital theory and cultural capital theory to justify the impact relationship between the variables, and we are well aware that the theory needs to be further deepened and improved, which is also the focus of our subsequent research work, we have updated the argumentative

formulation of public physical activity to personal physical activity in the hypothesis section, and we believe that your previous comments are very reasonable.

We hope that the revisions and improvements can satisfactorily address the concerns of the reviewers. We appreciate for Editor warm work earnestly, and hope that the correction will meet with approval. Once again, thank you very much for your comments and suggestions.
