# Supplementary material for: Rural active aging as a development strategy: Nexus of physical exercise participation and multidimensional poverty in China
Source: PLoS One. 2025 Sep 2;20(9):e0331199. doi: 10.1371/journal.pone.0331199 (PMC12404369; doi:10.1371/journal.pone.0331199)
Supplement: Attachment — Submitted filename: Response letter.docx [file pone.0331199.s002.docx]

**Rural Active Aging as Development Strategy: Quantifying Exercise’s Multidimensional Poverty Reduction Effects**

Qi-fei Xia^1^, Shu Xuan^2^, Guo-you Qin^3*^

1 School of Physical Education, Ankang College, Ankang, China

2 School of Physical Education, Hunan University, Changsha, Hunan, China

3College of Physical Education, Hanjiang Normal University, Shiyan, China

* Correspondence: Prof. Guo-you Qin,

Email: [qinguoyou22@163.com](mailto:qinguoyou22@163.com)

**Response Letter to Reviewer’s Comments**

**Comments from the Editor:**

Thank you for submitting your manuscript to PLOS ONE. After careful consideration, we feel that it has merit but does not fully meet PLOS ONE’s publication criteria as it currently stands. Therefore, we invite you to submit a revised version of the manuscript that addresses the points raised during the review process.

Reponse: We thank all the editors and reviewers for their valuable comments and suggestions.-We have carefully revised the manuscript to enhance its clarity and facilitate the understanding of the readers. Our point-to-point responses are presented in the following We-hope that the revision would satisfactorily address the comments and concerns of the editors and reviewers.

Response Letter

Dear Editor:

Thank you for your letter and comments concerning our manuscript entitled “Rural Active Aging as Development Strategy: Quantifying Exercise’s Multidimensional Poverty Reduction Effects” (Manuscript ID: PONE-D-25-1951), Those comments are all valuable and very helpful for revising and improving our paper. According to the Editor/Reviewer’s comments. we have made extensive modifications to our manuscript. In this revised version. Our response is given in normal front and changes/additions to the manuscript are given in the red text. the detailed corrections are listed below.

I hope that the revised manuscript for publication in *PLOS One*. Thank you again for your help with the manuscript. I am more than happy to provide any further information you may need.

**Responses to Reviewer # 1:**

*Q1: Abstract
The abstract is well-written but lacks focus in logically presenting the research findings and recommendations. For example:" Physical exercise has a significant poverty-reducing effect, with a multifaceted impact….."
This statement is too vague. How does physical exercise reduce poverty, and in what ways is the impact multifaceted? This is not sufficiently addressed, leaving the reader uncertain about what aspects of poverty are being discussed. The authors must explicitly state the pathway through which physical exercise contributes to multidimensional poverty reduction.*

ANSWER: We sincerely appreciate the valuable comments. Based on your suggestions, we have revised the research results and suggestions. We adhere to the standardization of abstract elements. The so-called conclusion is to review the main findings and significance of the research and may propose future research directions or suggestions. During the revision process, we expanded and supplemented the multiple aspects in which physical exercise affects multi-dimensional poverty. Specifically, the alleviation of multi-dimensional poverty by physical exercise is reflected in the enhancement of health levels, economic conditions, living standards, and subjective perception dimensions. Based on this modification, we also conducted robustness tests on the multi-dimensional poverty decomposition dimensions respectively in the following text to make the research results more scientific and rigorous. Furthermore, in the conclusion section, we have also adjusted the language logic of the mediating mechanism (social capital and cultural capital) and the moderating effect (network service usage and public service level), clarifying the internal mechanisms contained therein. Through modification, the research results and significance can be better presented, allowing readers to have a clearer understanding of the detailed process of this study. We hope that the revisions and improvements can satisfactorily address the concerns of the reviewers. The specific details of the revision can be found in lines 24 to 46 on the first page of the manuscript. Specific modification contents:

**Results**

This study demonstrates that participation in physical exercise significantly alleviates multidimensional poverty among elderly rural households. The result remains robust across a suite of validation strategies, including full-model estimations, propensity score matching, and instrumental variable methods. Social capital and enhanced cultural capital serve as mediating pathways, accounting for 10.86% and 33.14% of the total effect, respectively. Moreover, the impact of exercise on poverty reduction is significantly moderated by access to internet services and the quality of public services—both of which amplify the benefits of exercise participation. The poverty-alleviating effect is particularly pronounced among women, unmarried individuals, and residents in eastern regions.

**Conclusion**

Physical exercise offers a viable strategy for mitigating multidimensional poverty, with observable benefits across subdimensions such as health, income, living standards, and subjective wellbeing. These benefits are partly driven by the accumulation of social capital and improvements in cultural capital. Enhanced digital connectivity and public service provision further strengthen the role of exercise in poverty reduction.

**Recommendations**

We recommend improving rural sports infrastructure, integrating physical activity with agricultural labour through a “healthy labour” model, and launching targeted exercise-based poverty alleviation programs for vulnerable elderly populations. National initiatives such as “Healthy Villages” should be promoted to encourage widespread participation in physical activity.

(*The specific details of the revision can be found in lines 24-46 on the first page of the manuscript*)

*Q2: 2. Introduction*

*The introduction presents a strong case but does not clearly articulate the knowledge gap. What is the central problem? Are elderly individuals not engaging in exercise, or is multidimensional poverty particularly pronounced among them? Could it be that multidimensional poverty itself affects exercise frequency? Furthermore, why are internet access and social capital not considered? All elements of the theoretical framework should be integrated into this section to demonstrate how they logically connect to the research problem.*

ANSWER: Thanks for the comment. We are very grateful that you have carefully read our manuscript and made valuable suggestions for our introduction part. We clarified the core issue of this research in your suggestion. Firstly, our research elaborates in the first paragraph of the article on the current practical problem of multi-dimensional poverty among the elderly in rural areas and what means or approaches might be needed to solve this problem, triggering readers' thinking. Secondly, by reviewing the literature, we found that multi-dimensional poverty among the elderly in rural areas is influenced by both subjective and objective conditions. In this regard, physical exercise, as a low-cost and high-efficiency intervention method, offers the possibility. Currently, there are relatively few studies on the relationship between physical exercise and multi-dimensional poverty. Therefore, the research was explored from two aspects: First, living standards and economic conditions are sub-dimensions of multi-dimensional poverty. Through literature review, we have found that physical exercise plays a positive role in these two aspects. Second, there are numerous arguments regarding the connection between health levels and subjective feelings and physical exercise in this regard. In the manuscript, we mainly added three unresolved core issues, considered the problems of the mediating mechanism and the moderating effect, and incorporated all the elements of its theoretical framework into the core issue framework, which is mainly divided into the following three points: 1) The mechanism of poverty reduction through movement is ambiguous. The specific aspects of poverty reduction have not been discovered in previous studies, and there is a lack of exploration of the internal transmission mechanism. 2) Under the digital wave, what differentiated poverty reduction effects will the use of network services enable rural elderly people to access the Internet and participate in physical exercise? And how the development of public service levels, which provides more opportunities for participation in exercise, will affect the effect of poverty reduction are the key issues that need to be urgently addressed. 3) Due to the individual and regional differences, is there a structural change in the impact of physical exercise on multi-dimensional poverty? In conclusion, we have made modifications to present the problems that the research institute aims to solve more intuitively and clearly. Furthermore, to facilitate readers' understanding and to assess the actual contribution of this study, we have supplemented the marginal contribution of the manuscript in the introduction section: First, previous studies have focused on the one-dimensional poverty view of income, while our study, based on the capacity theory and the multi-dimensional poverty theory, has tentatively analyzed the mechanism of poverty reduction through movement from multiple perspectives, filling the existing research gap. Second, the influence of the mediating mechanism and the moderating effect was verified through various testing methods, enriching the theoretical basis of the research. Thirdly, through heterogeneity test analysis, this actual survey can better formulate effective policies based on local conditions, highlighting the comprehensive value and function of physical exercise. We hope that the revisions and improvements can satisfactorily address the concerns of the reviewers. The specific details of the revision can be found in lines 91-120 on pages 5 and 6 of the manuscript.

Specific modification contents:

While existing research has confirmed the poverty-alleviation effects of physical exercise [13], several critical gaps remain. First, the mechanisms underlying this effect are insufficiently understood, particularly regarding the mediating roles of social and cultural capital. Second, the differential impacts of exercise under the digital wave—such as disparities in access to internet services—and the moderating role of public service provision lack empirical validation. Third, variations in poverty reduction outcomes across regions with different resource endowments and development levels have yet to be systematically explored.Although previous studies have examined the influence of exercise on health [14], quality of life [12], and public needs [15] among rural populations, little attention has been paid to multidimensional poverty among elderly rural residents—a particularly vulnerable group—and the role of physical exercise in alleviating it.

To address these gaps, this study uses data from the China Family Panel Studies (CFPS) and applies the Alkire-Foster (A-F) dual cutoff method to measure the multidimensional poverty index of elderly members in rural households. The analysis is conducted at the household level to investigate the impact and mechanisms of physical exercise on multidimensional poverty. Our findings aim to offer both theoretical insights and empirical evidence to support long-term poverty alleviation and rural revitalization.This study makes several potential contributions. First, it moves beyond the income-centric view of poverty to reveal how physical activity enhances individual capabilities, thereby enriching the theoretical landscape of sport-based poverty alleviation. Second, building on multidimensional poverty and capability theories, we empirically examine how physical exercise reshapes social networks and expands cultural capital to reduce poverty, and how digital inclusion and public service levels moderate this relationship. These findings provide actionable insights for addressing complex poverty issues in developing countries. Finally, by exploring the heterogeneous effects of exercise across demographic and regional groups, the study offers valuable policy guidance for localized poverty-reduction strategies.

(*The specific details of the revision can be found in lines 91-120 on pages 5 and 6 of the manuscript.*)

*Added references*

Oettle, L., & Greiner, J. (2025). Coping with Poverty and Social Exclusion: Promoting Capabilities Through Long‐Term Sports Participation. Social Inclusion, 13.

*Q3: 3. Literature Review*

*What is the theoretical foundation of the study? Upon what framework are the hypotheses built? The authors appear to rely primarily on empirical literature rather than an established theory that supports the idea that exercise can reduce multidimensional poverty. Additionally, the mediating and moderating variables should be theoretically justified.*

ANSWER: Thanks for the comment. We are very grateful to you for providing valuable reference suggestions for the literature review section. Based on your suggestion, we have searched a large number of literature materials to provide the corresponding theoretical basis for poverty reduction through exercise. We find that the modern development economist Amatia Sen introduced the concept of "capability" to analyze poverty. This theory emphasizes the combination of various functions that individuals can achieve and establishes three key and interrelated elements: capability, function and transformation factor. Among them, ability represents the opportunity or freedom for an individual to achieve valuable functions - that is, the existence and behavior that they have reasons to cherish. Function refers to the actual goals achieved by an individual, such as employment, participation in community activities or maintaining good health. The transformation factor regulates this relationship and influences how resources and opportunities are transformed into realized functions. Physical exercise, as an extensible and important core competence, can systematically alleviate the complex deprivation of multi-dimensional poverty, which has been demonstrated in previous literature. (Oettle, L., & Greiner, J. (2025). Coping with Poverty and Social Exclusion: Promoting Capabilities Through Long‐Term Sports Participation. Social Inclusion, 13.)

In terms of the influencing mechanism, social capital and cultural capital may be important functional factors. The poverty reduction efficacy of physical exercise is manifested as a chain reaction of "capacity production - capital transformation - poverty breakthrough". At the level of social capital, we introduce Bourdieu's theory of social capital construction and construct social capital variables from two levels: individual social capital and collective social capital. For cultural capital, we introduce Bourdieu's theory of cultural capital and describe it based on an individual's educational attainment and whether they read or not. Overall, the application of these theories has laid a good theoretical foundation and reference basis for variable construction. Through empirical tests, it can further reveal that social capital and cultural capital, as the actual goals achieved by individuals, can give full play to the functions of social network relationships and knowledge literacy in the process of training poverty reduction, and play an important role in alleviating multi-dimensional poverty.

In the feasibility theory, the transformation factor is usually manifested as the influence of external factors, and is manifested as how to regulate the transformed functions to be realized. We can consider that the use of network services and the level of public services, as important external intervention factors, may be moderated and affected by the access to digital technology and the improvement of the level of public services in the process of poverty reduction through exercise for the elderly of household farmers.

Based on this, our research hypotheses are mainly established on the basis of the theoretical analysis framework of feasible capabilities, providing theoretical support for empirical research. The specific details of the revision can be found in lines 160 and 176 of the manuscript.

Specific modification contents:

Drawing on Amartya Sen’s capability approach, this study conceptualizes poverty not merely as a lack of income, but as a deprivation of the freedoms to achieve valuable functionings—such as good health, stable income, a quality life, and a sense of self-worth [17]. This framework centres on three interrelated components: capabilities, functionings, and conversion factors [19,20,21,22]. Capabilities refer to the real opportunities individuals have to achieve outcomes they value; functionings are the realized states or activities, such as being employed, engaging in community life, or maintaining good health; conversion factors influence how resources and opportunities are transformed into actual achievements.Physical exercise—being low-cost and high in positive externalities—serves as an effective intervention to enhance individuals' capabilities and thus mitigate multidimensional poverty. Specifically, it can contribute through several pathways. 1.Health as a Critical Factor: health plays a foundational role in labor capacity. According to human capital theory, health constitutes a critical form of human capital, and maintaining good physical condition is a prerequisite for engaging in productive work and generating income [23].

(*The specific details of the revision can be found in lines 160 and 176 on the manuscript*)

*Added references*

Robeyns, I. (2005). The capability approach: A theoretical survey. Journal of Human Development, 6(1), 93–117.https://doi.org/10.1080/146498805200034266

Robeyns, I. (2017). Wellbeing, freedom and social justice: The capability approach re‐examined. Open Book Publishers. https://doi.org/10.11647/OBP.0130

Sen, A. (2001). Development as freedom. Oxford University Press.

Sen, A. (2010). Inequality reexamined (15th ed.). Oxford University Press.

Sweetland, S. R. (1996). Human capital theory: Foundations of a field of inquiry. Review of educational research, 66(3), 341-359.

*Q4: 4. Data*

*4.1 A key issue is how physical exercise is operationalized. Considering the study focuses on elderly rural populations, using a broad dummy variable may not be appropriate, especially since the type of exercise is unspecified. It is crucial to clarify what kinds of exercise are included and how much is required to have a meaningful effect. Additionally, what are the criteria for exercise in terms of age groups? A more detailed description of the sample is warranted.*

*4.2 Regarding the Multidimensional Poverty Index (MPI), beyond the aggregate measure, the authors should disaggregate poverty into its key components—health, education, and quality of life—to ensure the robustness of the results.*

*4.3 The empirical strategy should be made more reader-friendly. Complex methodologies need to be explained clearly for broader accessibility. Further methodological concerns include the treatment of endogeneity and reverse causality—how were these addressed? Additionally, it is recommended to bootstrap the Sobel test for more accurate standard errors, which is a crucial step in ensuring the validity of the findings.*

ANSWER: We appreciate your summary of the manuscript and encourageing comment. We have made detailed modifications in this part. In Section 4.1, a key question is why physical exercise is a dummy variable. In this section, we have added the specific types of exercise and why the exercise frequency was chosen as the core independent variable. Furthermore, after reviewing a large number of literature materials, we found that the American College of Sports Medicine (ACSM) recommends that people engage in moderate physical activity at least three times a week or more, and each session lasts for 30 minutes or longer. However, there are a large number of missing values in the exercise time variable of the CFPS questionnaire data. Continuing to select it may also cause bias in the research results. Therefore, referring to the variable division ideas of predecessors, we consider an average weekly exercise frequency of ≥3 times as the existence of exercise participation behavior, and assign a value of 0 for the contrary. The basis for such a division is to meet the physical activity requirements of ACSM in the United States and the actual needs of the research. For details, please refer to the Variable Modification section. Furthermore, we have added sample descriptions of elderly household farmers aged 45 and above to present a clearer research process. The specific details of the revision can be found in lines 287, 353 and 367 of the manuscript.

Specific modification contents:

1. The core explanatory variable is participation in physical exercise among elderly individuals in rural Chinese households. The CFPS dataset captures a wide range of physical activities, including periodic exercises such as walking, running, jogging, and hiking; ball games such as football, basketball, and table tennis; and water-based activities like swimming, sailing, and diving. According to prior research [55], total physical activity should be assessed across dimensions of frequency, duration, and intensity. The American College of Sports Medicine (ACSM) recommends engaging in at least three sessions of moderate-intensity physical activity per week, each lasting 30 minutes or more [56]. In this study, participation in physical exercise is proxied by exercise frequency. Due to substantial missing and abnormal values in the exercise duration variable across CFPS waves, we use self-reported frequency as the indicator. Based on the survey question “How many times did you exercise in the past week?” and following Liu [12], we construct a binary variable: individuals reporting exercise ≥3 times per week are classified as participating in physical exercise (coded as 1); otherwise, they are coded as 0.
2. The sample includes rural elderly individuals aged 45 and above, with personal and family databases merged using family sample identification codes. Relevant variables were selected, and the data were cleaned, resulting in a final sample of 6,233 elderly rural households, with 3,876 from 2018 and 2,357 from 2022. Public service level data were sourced from the China Statistical Yearbook, while instrumental variable data came from the China Sports Industry Statistical Yearbook.

(*The specific details of the revision can be found in lines 287, 353 and 367 on the first page of the manuscript*)

*Added references*

Guo J, Guan L, Fang L, et al. Depression among Chinese older adults: A perspective from Hukou and health inequities[J]. Journal of Affective Disorders, 2017, 223: 115-120.

American College of Sports Medicine, The recommended quantity and quality of exercise for developing and maintaining cardiorespiratory and muscular fitness in healthy adults.  Med Sci Sports Exerc 1990;22 (2) 265- 274

In Section 4.2, we decomposed poverty into key components for robustness tests. The results remained significant, confirming the impact of physical exercise on multi-dimensional poverty sub-dimensions and the robustness of the research findings. The specific details of the revision can be seen in Table 9. Specific modification contents:

**Multidimensional Poverty Decomposition:** As shown in Table 9, Model indicates that physical exercise significantly improves health status, economic conditions, living standards, and subjective wellbeing. These results suggest that exercise contributes to enhanced functional ability and reduced depressive symptoms, increased consumption, reduced daily living expenses, and improved self-perception. Collectively, these improvements further validate the poverty-reducing effects of physical activity.

In terms of methodological issues, we adopted the propensity score matching method to solve the problem of sample selection bias, and utilized the instrumental variable method to handle the endogeneity problem caused by reverse causality as much as possible. Before solving these problems, we used DWH to test whether endogeneity problems existed or not, making the research design clearer and more transparent. Furthermore, we added more accurate standard errors in the sobel test to ensure the robustness of the research results. The specific details of the revision can be seen in Table 5.

*Q5: 5. Results*

*The presentation of results is unclear and confusing. Each model should be explained separately, including a detailed explanation of the columns in each results table. The findings should be interpreted within the context of existing literature to highlight contributions and deviations. Most critically, the causal pathway between physical exercise and multidimensional poverty must be better established. Otherwise, the findings risk appearing too simplistic.*

ANSWER: Thanks for the comment. According to the reviewers' suggestions, we meticulously modified the presentation of each result, such as the regression coefficients and *p*-value significance of each column of results in each model, and provided detailed explanations of the obtained results. Meanwhile, in order to better explain the research results, we supplemented the corresponding literature to further demonstrate the influence relationship between strengthened physical exercise and multi-dimensional poverty, and analyzed the differences from the results of previous studies, as well as the actual contributions obtained from the results of this study. In this regard, we have also made supplements in terms of mediating mechanisms and moderating effects in order to better combine the similarities and differences between the research results and those of previous studies. The specific details of the modification can be found in lines 466 and 482. Specific modification contents:

Empirical studies have shown that long-term engagement in physical activity enhances individuals’ capacity to manage daily life, fosters a sense of social belonging and confidence, and facilitates positive physical and psychological transformations—factors that contribute to poverty reduction [13]. Our findings align with this evidence, confirming the poverty-alleviation effect of physical exercise. In Model (2), after controlling for individual characteristics, the association between exercise participation and multidimensional poverty among elderly rural residents remains statistically significant (*β* = –0.0146, *p* < 0.001). Among individual-level covariates, gender, age, and marital status are all significantly associated with multidimensional poverty.Model (3) adds regional characteristics. Although regional effects are not statistically significant, the negative coefficient suggests that elderly individuals in less developed western regions may face more severe multidimensional poverty.To test robustness, we conduct regressions for different years. Models (4) and (5) indicate that the poverty-reducing effect of physical activity has strengthened over time (*β* = –0.0171, *p* < 0.001; *β* = –0.0508, *p* < 0.05), further supporting the stability of our results (see Table 4).

We employed hierarchical stepwise regression to preliminarily test the mediating mechanisms. In Model (1), physical exercise shows a significant negative association with multidimensional poverty *(β* = –0.0149, *p* < 0.001). In Models (2) and (4), the coefficients for social capital and cultural capital are both significantly positive, indicating that exercise participation promotes the accumulation of these forms of capital among elderly rural residents.Model (3) reveals a significantly negative coefficient, suggesting that physical activity alleviates poverty through enhanced social capital. Prior studies indicate that even informal or leisure-based physical activities can help individuals expand their social capital [63], enabling rural households to access poverty-reduction resources through broader social networks, thereby reducing the risk of poverty recurrence [64]. Similarly, Model (5) shows a significant negative coefficient, indicating that exercise mitigates multidimensional poverty through the enhancement of cultural capital. Evidence suggests that individuals who regularly engage in exercise tend to acquire more health-related knowledge and develop stronger health values and behavioral norms—key components of cultural capital [65]. When cognitive patterns, habitual behaviors, and resource access capabilities reach a certain threshold, they may help break the intergenerational transmission of poverty [66].

(*The specific details of the revision can be found in lines 466 and 482 on the manuscript*)

*Added references*

Oettle, L., & Greiner, J. (2025). Coping with Poverty and Social Exclusion: Promoting Capabilities Through Long‐Term Sports Participation. Social Inclusion, 13.

Youhua, W. A. N. G., Ming, L. E. I., & Yuxiao, G. A. O. (2023). Community-Level Social Capital and Physical Exercise of Urban Residents An Empirical Analysis Based on the General Multilevel Linear Model. Journal of Shanghai University of Sport, 47(6), 33-41.

Wang, J., Xiao, H., & Liu, X. (2022). The impact of social capital on multidimensional poverty of rural households in China. International Journal of Environmental Research and Public Health, 20(1), 217.

Mudd, A. L., Oude Groeniger, J., Bal, M., Verra, S. E., van Lenthe, F. J., & Kamphuis, C. B. (2023). Testing conditionality with Bourdieu's capital theory: How economic, social, and embodied cultural capital are associated with diet and physical activity in the Netherlands. SSM-Population Health, 22, 101401.

Shubin, S. (2010). Cultural exclusion and rural poverty in Ireland and Russia. Transactions of the Institute of British Geographers, 35(4), 555-570.

*Q6: 6. Conclusion*

*The conclusion is notably weak. It lacks practical policy recommendations and fails to acknowledge the study's limitations or suggest directions for future research.*

*A strong conclusion would:*

*• Emphasize the practical implications of promoting physical exercise among elderly rural populations,*

*• Discuss the feasibility of interventions,*

*• And acknowledge constraints such as cultural factors, health limitations, or infrastructure gaps.*

ANSWER: Thanks for the comment. First of all, we are very grateful for your reference suggestions in the conclusion section. During the revision process, we sorted out the logical expression of the research conclusion, especially explaining the causal effect of exercise poverty reduction, which is specifically manifested in the multi-faceted impact of physical exercise on multi-dimensional poverty, such as health status, economic level, etc. This is also basically consistent with the results obtained in our article.

Secondly, in this study, we added the limitations of the research and the proposed directions for the future in the conclusion section, which was also mentioned in the comments of Reviewer 2. In this part, we believe that the limitations include the following aspects: First, due to data limitations, using only the frequency indicator of physical exercise is somewhat monotonous and cannot fully interpret the actual connotation of physical exercise. Subsequently, more comprehensive and updated data will be explored and matched for verification. Second, the research period was only two years. Subsequently, quasi-natural experiments need to be adopted to explore the dynamic effects of poverty reduction through sports, which can provide more sufficient empirical evidence. Finally, it may be singular for the study to only select the elderly of household farmers as the survey group, and it is necessary to expand the differential effects of exercise in poverty reduction among different age groups in different regions.

Finally, we supplemented policy recommendations after the research conclusion. In this part, we discussed what kind of exercise intervention should be adopted for the elderly in family farmers to achieve poverty alleviation, emphasizing the practical significance of poverty reduction through exercise. Meanwhile, we have also put forward some specific and practical suggestions, such as establishing a "healthy labor model" that combines physical exercise with agricultural production, integrating it with existing elderly care institutions and home-based elderly care services, strengthening social sports guidance and social service activities, and reducing economic poverty and social exclusion caused by health problems. And establish community fitness challenge competitions and health reward mechanisms to encourage the elderly and their families to participate in exercise together and enhance the health awareness of the elderly in rural areas. These measures can provide some valuable references for consolidating the achievements of poverty alleviation. The specific details of the modification can be found in lines 538-549.

Specific modification contents:

This study investigates the impact and mechanisms of physical exercise participation on multidimensional poverty among elderly rural households. The key findings are as follows:Exercise significantly reduces multidimensional poverty. Regular participation improves health outcomes, stabilizes economic conditions, enhances living standards, and fosters subjective wellbeing, offering an effective pathway out of poverty for elderly rural populations.Social and cultural capital serve as key mediators. Physical activity strengthens social networks and enhances individual knowledge and capabilities, thereby providing multiple channels through which poverty can be alleviated.Digital access and public service provision amplify the effect. Use of internet services and improved public infrastructure increase opportunities for rural elderly individuals to engage in exercise, identifying critical areas for targeted policy support.Heterogeneous effects are observed. The poverty-reduction impact of exercise is particularly pronounced among women, unmarried individuals, and those living in eastern regions. These findings highlight the need for tailored, context-specific exercise interventions to prevent the transmission of poverty across vulnerable groups.

Despite its contributions, this study has several limitations:(1) The CFPS dataset contains substantial missing values in the exercise duration variable, and the sample used is relatively limited. Future research should leverage larger and more diverse datasets for validation.(2) This study focuses on mediation and moderation mechanisms but does not explore the dynamic changes in the poverty-alleviation effects of exercise before and after China’s targeted poverty alleviation campaign. Future work could adopt quasi-natural experimental designs and multi-period difference-in-differences (DID) models to address this gap.(3) The study targets elderly rural households, and the sample may not fully represent other regions or demographic groups. As rural revitalization and agricultural modernization advance, the generalizability of the findings warrants further verification. Future research should incorporate additional variables and utilize panel data to better identify causal pathways and enhance the robustness of conclusions.

The findings of this study offer important policy insights for addressing multidimensional poverty among elderly rural households in China. First, efforts should be made to enhance the construction and maintenance of rural sports infrastructure. As physical exercise plays a significant role in alleviating poverty, governments should increase investment in public fitness equipment, walking trails, and open spaces in rural areas. Special attention should be given to improving the accessibility and usability of sports facilities in remote regions. In addition, an integrated system linking exercise and medical services should be established. This could include regular health screenings, exercise assessments, and real-time monitoring of elderly individuals’ physical conditions to ensure effective and safe participation.

Second, a “healthy labour” model that integrates physical exercise with agricultural production should be developed. Many rural elderly individuals continue to engage in lifelong manual labour, both as a source of income and as a way to sustain self-worth. Introducing light exercise elements into agricultural activities—for example, promoting stretching, walking, or calisthenics during work—can help mitigate health risks associated with physical labour. This approach may enhance productivity while fostering long-term health–labour balance.

Third, targeted exercise-based poverty alleviation initiatives should be implemented for elderly populations at risk of falling back into poverty. To prevent poverty driven by illness or vulnerability, programs should focus on disadvantaged, frail, and isolated elderly individuals. These interventions can be combined with nutritional support, health education, and healthcare subsidies to strengthen the health component of poverty alleviation policy. Integration with existing elderly care institutions and home-based care services, alongside increased provision of community sports guidance and social support, can reduce both economic hardship and social exclusion caused by poor health.

Fourth, the “Healthy Village” initiative should be promoted to encourage universal participation in physical activity. Nationwide fitness campaigns hold significant potential for advancing rural revitalization. Through coordinated efforts by government, community organizations, and private enterprises, regular fitness events, health screenings, and educational programs can be organized. Community fitness challenges and incentive schemes may help mobilize elderly individuals and their families to adopt active lifestyles, enhancing awareness of senior health and fostering a culture of collective wellbeing in rural society.

(*The specific details of the revision can be found in lines 538-549 on the first page of the manuscript*)

**Responses to Editor # 2:**

*Q1: - The literature in the Lit. Rev. section should be updated further. Some citations are quite dated.*

ANSWER: Thanks for the comment. We are extremely grateful for your valuable suggestions. According to your suggestion, we have replaced some of the outdated literature in the literature review. The replacement time mainly comes from the references in the past five years. The specific replacement contents are as follows:

| 序号 | 时间 | 标题 |
| --- | --- | --- |
| 11 | 2022 | Xiao, X., Yu, Y., He, Q., Xu, D., Qi, Y., Ma, L., & Deng, X. (2022). Does regular physical activity improve personal income? Empirical evidence from China. Nutrients, 14(17), 3522. |
| 21 | 2025 | Wen, Z., Kim, Y., & Choi, Y. (2025, March). Effects of Exercise Program on Mental, Pulmonary, and Cardiovascular Health of Elderly Men with Acquired Severe Physical Disabilities: A Retrospective Study. In Healthcare (Vol. 13, No. 6, p. 597). MDPI. |
| 26 | 2024 | Velaithan, V., Tan, M. M., Yu, T. F., Liem, A., Teh, P. L., & Su, T. T. (2024). The association of self-perception of aging and quality of life in older adults: a systematic review. The Gerontologist, 64(4), gnad041. |
| 27 | 2022 | Zhao, W., Hu, P., Sun, W., Wu, W., Zhang, J., Deng, H., ... & Liu, X. (2022). Effect of physical activity on the risk of frailty: A systematic review and meta-analysis. PloS one, 17(12), e0278226. |
| 33 | 2022 | Veitch, J., Ball, K., Rivera, E., Loh, V., Deforche, B., Best, K., & Timperio, A. (2022). What entices older adults to parks? Identification of park features that encourage park visitation, physical activity, and social interaction. Landscape and Urban Planning, 217, 104254. |
| 39 | 2019 | Horolets, A., Stodolska, M., & Peters, K. (2019). Natural environments and leisure among rural–to–urban immigrants: an application of Bourdieu's concepts of habitus, social and cultural capital, and field. Leisure sciences, 41(4), 313-329. |
| 47 | 2024 | Zhang, H. (2024). The Impact of Community Sports Public Services on Public Satisfaction. Revista de Psicología del Deporte (Journal of Sport Psychology), 33(4), 127-135. |

*Q2: - 'Limitations and future agenda' should be expanded upon further and moved to the 'Conclusions' section.*

ANSWER: Thanks for the comment. Based on your suggestion, we have moved the research limitations and future research prospects to the "Conclusions" section. Thank you very much for your valuable suggestions.

*Q2: In order to strengthen generalisability, I would expand the reflection on why one would expect the effects to vary in other populations (e.g. young people or urban populations) or geographical contexts (e.g. countries with different welfare systems, sports infrastructures, or cultural norms regarding exercise).*

ANSWER: Thanks for the comment. Thank you very much for your valuable suggestions. According to the reviewers' requests, we have supplemented policy recommendations after the research conclusion. We believe that through the research on the poverty reduction mechanism of the elderly movement of household farmers, it can provide reference suggestions for other groups or geographical environments in developing countries or other regions. In the policy recommendations section, we have also added different exercise intervention measures for the elderly in family farmers, as well as targeted suggestions and thoughts on how to combine agricultural production, participation in social activities, and leisure interaction. We hope that the revisions and improvements can satisfactorily address the concerns of the reviewers.

We appreciate for Editor warm work earnestly, and hope that the correction will meet with approval. Once again, thank you very much for your comments and suggestions.
