# Supplementary material for: Rural active aging as a development strategy: Nexus of physical exercise participation and multidimensional poverty in China
Source: PLoS One. 2025 Sep 2;20(9):e0331199. doi: 10.1371/journal.pone.0331199 (PMC12404369; doi:10.1371/journal.pone.0331199)
Supplement: Attachment — Submitted filename: Response_letter_auresp_4.docx [file pone.0331199.s006.docx]

**Rural Active Aging as Development Strategy: Quantifying Exercise’s Multidimensional Poverty Reduction Effects**

Qi-fei Xia^1^, Shu Xuan^2^, Guo-you Qin^3*^

1 School of Physical Education, Ankang College, Ankang, China

2 School of Physical Education, Hunan University, Changsha, Hunan, China

3College of Physical Education, Hanjiang Normal University, Shiyan, China

* Correspondence: Prof. Guo-you Qin,

Email: [qinguoyou22@163.com](mailto:qinguoyou22@163.com)

**Response Letter to Reviewer’s Comments**

**Comments from the Editor:**

1.*Thank you for submitting your manuscript to PLOS ONE. After careful consideration, we feel that it has merit but does not fully meet PLOS ONE’s publication criteria as it currently stands. Therefore, we invite you to submit a revised version of the manuscript that addresses the points raised during the review process.*

Reponse: We thank all the editors and reviewers for their valuable comments and suggestions.-We have carefully revised the manuscript to enhance its clarity and facilitate the understanding of the readers. Our point-to-point responses are presented in the following We-hope that the revision would satisfactorily address the comments and concerns of the editors and reviewers.

2.*We kindly ask you to provide a detailed and thorough response to the comments raised by the reviewer in their latest evaluation. It is essential to carefully address all the points highlighted, making the necessary revisions and offering comprehensive clarifications. This approach will significantly improve the quality of your work and increase the likelihood of its unconditional acceptance.*

Reponse: We thank you very much for the opportunity to revise the manuscript, which we take very seriously, and we have always found the suggestions provided by the reviewers to be very helpful in terms of the quality of our work, to which we have responded in a concise, point-to-point, substantive manner, which we hope addresses the concerns of both the reviewers and the editors.

Response Letter

Dear Editor:

Thank you for your letter and comments concerning our manuscript entitled “Rural Active Aging as Development Strategy: Quantifying Exercise’s Multidimensional Poverty Reduction Effects” (Manuscript ID: PONE-D-25-1951), Those comments are all valuable and very helpful for revising and improving our paper. According to the Editor/Reviewer’s comments. we have made extensive modifications to our manuscript. In this revised version. Our response is given in normal front and changes/additions to the manuscript are given in the blue text. the detailed corrections are listed below. The letter of reply has been placed in the annex.

I hope that the revised manuscript for publication in *PLOS One*. Thank you again for your help with the manuscript. I am more than happy to provide any further information you may need.

Dear Reviewer:

Thank you for your letter and comments concerning our manuscript entitled “Rural Active Aging as Development Strategy: Quantifying Exercise’s Multidimensional Poverty Reduction Effects” (Manuscript ID: PONE-D-25-1951), Those comments are all valuable and very helpful for revising and improving our paper. We are very sorry that we have not been able to address your concerns well in each revision, and in this revision process we have carefully revised all of the sections from the Abstract, Methodological Strategy, and Results and Discussion sections. We would like to explain that we think about revision options immediately after receiving your suggestions in each case, and this manuscript is crucial for us because we need to conduct title reviews or other similar studies this year, which makes us respond more quickly or hurriedly. But don't worry, we have reviewed a lot of literature during each revision process, and we are very grateful for your professional advice, which has helped us to improve the quality of the manuscript, after we have given it much thought and hope to reply to you in the fastest time possible. In order to better complete this revision, we have provided a revised version (Word) version of the manuscript, and we have also added revised annotations inside the manuscript, so that you can better access, and we have also provided a letter of reply to your revised comments, according to your requirements, briefly explaining the contents of the revision, and hope to solve your concerns. Thank you again for your review. The letter of reply has been placed in the annex.

I hope that the revised manuscript for publication in *PLOS One*. Thank you again for your help with the manuscript. I am more than happy to provide any further information you may need.

**Responses to Reviewer # 1:**

*Comments Q1: Title Recommendation:
The revised title should not include the phrase “An Empirical Analysis Based on CFPS Data”, especially since most readers may not be familiar with CFPS. Below are two conditional title suggestions, depending on your chosen methodological approach:*
① *If you decide to use an IV method like PSM, with a well-identified instrumental variable as the main model to estimate the effect of physical exercise on multidimensional poverty (and use the baseline model only for robustness checks):
Suggested Title:
Rural Active Aging as a Development Strategy: Impact of Physical Activity Participation on Multidimensional Poverty in China*
② *If you choose to retain the baseline regression as your main model and remove the robustness checks conducted using the IV approach (which, in my view, is more methodologically sound):
Suggested Title:
Rural Active Aging as a Development Strategy: Nexus of Physical Activity Participation and Multidimensional Poverty in China.*

ANSWER: Thank you very much for your suggestion, we have addressed this issue as per your request, we have selected suggested option 2 and revised the title to: *Rural Active Aging as a Development Strategy: Nexus of Physical Exercise Participation and Multidimensional Poverty in China.* We hope our revision addresses your concerns. See manuscript title for specific changes.

*CommentsQ2：Note: Please carefully consider whether “physical activity” or “physical exercise” is the most appropriate term for your study. I believe physical exercise may be more accurate.*

ANSWER: Thanks for the comment. Following your suggestions, We have replaced the full text with the expression “Physical exercise” to be more consistent with the standard.

*CommentsQ3: Conceptual Framing and Hypotheses:
I recommend that the conceptual framing focuses on physical exercise and the mediating role you intend to test. Given the limited and unclear data coverage of public service and internet use variables, it would be better to avoid emphasizing these as moderators in your hypotheses.
Specifically, Hypothesis 31a remains unclear. The term “internet use” is too vague, lacking clarity on what aspects of internet use are being tested. Hypotheses must be specific and testable—not included for the sake of it. I strongly suggest removing this hypothesis. However, you may still analyze this moderating effect in the results section, provided it is carefully presented in the methods..*

ANSWER: Thank you very much for your review suggestion, we have addressed this issue based on your suggestion: we have removed the conceptual framework and research hypothesis of the moderating effect (public service level and internet service use) in consideration of the rigour of the study, the reason for the removal of this section is, as you mentioned, that the measurement of moderating effect section of the study is not specific enough, and that there may be an imposed relationship of the variables, which is taken into account by us and removed. We have taken this into account and removed it. Thank you very much for your suggestion. The deleted sections have been underlined for ease of reference.

.

*Comments Q4：Methods Section Structuring:
Here’s how you could improve the methods section under both scenarios:
If following Option 2 (Baseline approach):
2.2 Analytical Strategy
2.2.1 OLS:
Explain how you use OLS to analyze the association between physical exercise and multidimensional poverty. Briefly describe why OLS is appropriate. Additionally, explain how interaction terms (e.g., for internet use or public services) were incorporated to test for moderation, if you choose to retain this analysis.
2.2.2 Stepwise regression or KHB decomposition:
You should select only one of these techniques. Clearly justify your choice in a brief and intelligent manner. There is no need for a dedicated subsection to explain this—just integrate the rationale within the methods.
This structure will make your methods section more focused and clearer.*

ANSWER: Thank you very much for your review suggestion, according to your request, we have solved this problem: firstly, in the methodological strategy section, we briefly introduced the principle of the application of the OLS model, since the dependent variable multidimensional poverty is a continuous variable, and physical activity is a categorical variable, the results can be better estimated by using the OLS model; secondly, in the section of the model regression results, we have supplemented the information on how to analyse the correlation between the two by using the OLS model that is, for every unit increase in the independent variable (physical activity), the dependent variable (multidimensional poverty) will decrease by one unit accordingly. **The modifications are described in sections 3.3 and 4.1 of the methodological strategy.** Finally, we mediation effect only selected stepwise regression method to test, deleted the KHB decomposition method and the applicable principles to avoid the excessive complexity of the model.

*Comments Q5: Results and Conclusion:
The results section should logically follow the selected methodological approach, and the conclusion should directly reflect the key findings and chosen analytical strategy.*

ANSWER: Thank you very much for your review suggestion, we solved the problem: the results section follows the methodological sequence of OLS model regression → mediation effect analysis → robustness test → heterogeneity analysis; in the conclusion section, we deleted the non-essential expressions and supplemented the analytical strategy, which corresponds to the results section and presents the results more directly. **See manuscript section 6.Conclusion**.

We hope that the revisions and improvements can satisfactorily address the concerns of the reviewers. We appreciate for Editor warm work earnestly, and hope that the correction will meet with approval. Once again, thank you very much for your comments and suggestions.
